# Light Regulation of Chlorophyll and Glycoalkaloid Biosynthesis During Tuber Greening of Potato *S. tuberosum*

**DOI:** 10.3389/fpls.2020.00753

**Published:** 2020-06-30

**Authors:** Haruko Okamoto, Laurence J. M. Ducreux, J. William Allwood, Pete E. Hedley, Alison Wright, Vidyanath Gururajan, Matthew J. Terry, Mark A. Taylor

**Affiliations:** ^1^School of Biological Sciences, University of Southampton, Southampton, United Kingdom; ^2^Institute for Life Sciences, University of Southampton, Southampton, United Kingdom; ^3^Cell and Molecular Sciences, The James Hutton Institute, Dundee, United Kingdom; ^4^Branston Ltd., Lincoln, United Kingdom; ^5^B-hive Innovations Ltd., Lincoln, United Kingdom

**Keywords:** *S. tuberosum*, King Edward (KE), chlorophyll, glycoalkaloid, red/far-red light, blue light

## Abstract

Potato, *S. tuberosum*, is one of the most important global crops, but has high levels of waste due to tuber greening under light, which is associated with the accumulation of neurotoxic glycoalkaloids. However, unlike the situation in de-etiolating seedlings, the mechanisms underlying tuber greening are not well understood. Here, we have investigated the effect of monochromatic blue, red, and far-red light on the regulation of chlorophyll and glycoalkaloid accumulation in potato tubers. Blue and red wavelengths were effective for induction and accumulation of chlorophyll, carotenoids and the two major potato glycoalkaloids, α-solanine and α-chaconine, whereas none of these accumulated in darkness or under far-red light. Key genes in chlorophyll biosynthesis (*HEMA1*, encoding the rate-limiting enzyme glutamyl-tRNA reductase, *GSA*, *CHLH* and *GUN4)* and six genes (*HMG1*, *SQS*, *CAS1*, *SSR2*, *SGT1* and *SGT2*) required for glycoalkaloid synthesis were also induced under white, blue, and red light but not in darkness or under far-red light. These data suggest a role for both cryptochrome and phytochrome photoreceptors in chlorophyll and glycoalkaloid accumulation. The contribution of phytochrome was further supported by the observation that far-red light could inhibit white light-induced chlorophyll and glycoalkaloid accumulation and associated gene expression. Transcriptomic analysis of tubers exposed to white, blue, and red light showed that light induction of photosynthesis and tetrapyrrole-related genes grouped into three distinct groups with one group showing a generally progressive induction by light at both 6 h and 24 h, a second group showing induction at 6 h in all light treatments, but induction only by red and white light at 24 h and a third showing just a very moderate light induction at 6 h which was reduced to the dark control level at 24 h. All glycoalkaloid synthesis genes showed a group one profile consistent with what was seen for the most light regulated chlorophyll synthesis genes. Our data provide a molecular framework for developing new approaches to reducing waste due to potato greening.

## Introduction

Potato is the leading non-grain commodity in the global food system and the largest tuber food crop in terms of human consumption (Birch et al., 2012). However, potato tubers are one of the most perishable forms of fresh produce (WRAP, 2014). A major problem is that on exposure to light, tubers accumulate chlorophyll and turn green, an issue that effects all sectors of the supply and storage chains. There is a general assumption that tuber greening is associated with the accumulation of a group of plant defense compounds called glycoalkaloids (GAs), which are harmful for human consumption at high dosage (Nema et al., 2008). Despite the economic implications, the functional mechanisms underlying tuber chlorophyll and GA accumulation have not been well characterized.

Potato tubers contain a large number of starch-storing amyloplasts and, upon exposure to light, amyloplasts in the peripheral cell layers develop into chloroplasts that are capable of photosynthesis through the development of photosystems and their associated antenna proteins (Murajafras et al., 1994). The primary photosynthetic pigments in these proteins are chlorophyll *a* and *b* that are synthesized from the amino acid glutamate by a well-established biosynthesis pathway conserved across the plant kingdom (Mochizuki et al., 2010). The rate-limiting step in the pathway is the synthesis of 5-aminolevulinic acid (ALA) from glutamate in three enzymatic steps. Glutamate is activated to tRNA^Glu^ by glutamyl tRNA synthetase, which is converted to ALA by glutamyl-tRNA reductase (GluTR encoded by *HEMA* genes), and glutamate-1-semialdehyde-2,1-aminomutase (*GSA*). As tRNA^Glu^ is also the substrate for plastid protein synthesis, GluTR catalyzes the first committed step in the pathway and is subject to strong regulation (Brzezowski et al., 2015). Eight ALA molecules are then converted to protoporphyrin IX, which is committed to the synthesis of chlorophyll through the insertion of Mg^2+^ by magnesium chelatase. This enzyme is comprised of three subunits encoded by *CHLH*, *CHLI* and *CHLD* genes (Mochizuki et al., 2010) and is activated by the regulator GUN4 (Larkin et al., 2003). Previous experiments have established the *HEMA1*, *CHLH* and *GUN4* genes as part of a small group of key regulatory genes for the pathway in Arabidopsis (McCormac et al., 2001; McCormac and Terry, 2002; Matsumoto et al., 2004; Kobayashi and Masuda, 2016). This includes regulation via the phytochrome family of red (R) and far-red (FR) light photoreceptors in Arabidopsis seedlings (McCormac et al., 2001; McCormac and Terry, 2002; Kobayashi and Masuda, 2016) as well as in rice (Inagaki et al., 2015). In Arabidopsis, both phyA and phyB have been shown to contribute to induction of chlorophyll biosynthesis genes in R and FR light (McCormac and Terry, 2002; Stephenson and Terry, 2008). In addition, blue (B) light also induces chlorophyll biosynthesis, in this case via the cryptochrome family of photoreceptors, and cryptochromes 1 and 2 have both been shown to contribute to *HEMA1* and *CHLH* expression (McCormac and Terry, 2002; Stephenson and Terry, 2008). Although the effect of white light (WL) on inducing potato tuber greening has been studied previously (Tanios et al., 2018), the effect of B, R and FR light on chlorophyll accumulation is less well characterized and not at the level of gene expression. Evidence to date suggests that both B and R wavelengths are active in inducing chlorophyll in the cultivar Sebago (Petermann and Morris, 1985) with phytochrome proposed as the dominant photoreceptor (Morris et al., 1979).

Steroidal glycoalkaloids (GAs) have a neurotoxic effect on animal feeders and are common plant defense compounds in *Solanaceae* species, including potato (*S. tuberosum*), tomato (*S. lycopersicum*), and aubergine (*S. melongena*) (Piasecka et al., 2015). Genetic background is one of the primary factors in accumulation of GAs in potato tubers and modern varieties have been selected for their relatively low GA accumulation (Hellenas et al., 1995). In addition, environmental conditions such as light, drought, temperature and wounding have also been shown to induce steroidal GAs in potato tubers (Sinden et al., 1984; Griffiths et al., 1997; Friedman and McDonald, 1999; Bejarano et al., 2000) including in modern commercial varieties (Grunenfelder et al., 2006; Nahar et al., 2017). GAs are synthesized from a common primary metabolite, acetyl co-A, via the mevalonate pathway in the cytoplasm. Since this is the major pathway for cholesterol biosynthesis, enzymes are conserved across eukaryotes (Desmond and Gribaldo, 2009). In plants, this pathway branches for the synthesis of brassinosteroids and this biosynthetic branch is known to be regulated by phytochrome in Arabidopsis (Bancos et al., 2006). However, although an induction of GAs in potato tubers by light is well established (Percival, 1999), the mechanism of GA induction and its relationship to chlorophyll induction is not well understood.

In this research, we have investigated the effect of monochromatic B, R and FR light on the regulation of chlorophyll and GA accumulation in tubers of a commercial potato cultivar, King Edward (KE), which is particularly susceptible to tuber greening (Garnett et al., 2018), and demonstrate how the pathway is controlled via the induction of key biosynthesis genes for both chlorophyll and GA synthesis under these light conditions. Transcriptome analysis after 6 h and 24 h exposure to WL, B, and R showed induction of chlorophyll, carotenoid, GA, and phenylpropanoid biosynthesis, as well as photosynthesis-related genes. Our data thus provide a molecular framework for potato greening and open avenues for reducing agricultural and retail food waste caused by this process.

## Materials and Methods

### Plant Material and Culture Conditions

Potato cv. King Edward tubers were grown and harvested on behalf of Branston Ltd., in the summer 2016 and 2017 in Lincolnshire. Tubers were kept in darkness in a refrigerator (Medical LSFSR288UK, Lec, Merseyside, United Kingdom) monitored by a data logger and maintained at 3°C for up to 6 months until use. All experiments were repeated multiple times using both 2016 and 2017 harvests. Since all datasets were consistent, representative data are shown in each case. All tubers were washed in darkness and equilibrated to ambient temperature before being placed in a plant growth cabinet (LED30-HL1; Percival, USA) at 18°C either in darkness or in constant light provided by light emitting diodes (LED). Wavelengths of B (470 nm), R (660 nm), and FR (730 nm) light with ± 10 nm bandwidth or WL (400 – 680 nm) were used and at least three biological replicates (*n* = 3) each consisting of individual tubers were harvested at each time point starting at time 0 and then every 24 h for up to 10 days. Chlorophyll and carotenoid analyses were performed immediately after the completion of each light exposure experiment using fresh material. The same tubers were then cored and the samples were freeze dried for RNA and glycoalkaloid extraction.

The tubers analyzed by array analysis were acclimatized at 18°C for 24 h in darkness before exposure to B or R light for 6 or 24 h or kept in darkness at 18°C before harvest. Tubers were cored and immediately freeze dried before RNA extraction.

### Chlorophyll and Carotenoid Analysis

Tuber skin tissues were peeled to a thickness of 1 mm, including the periderm, and three 5 mm diameter disks were harvested from each tuber skin tissue as technical replicates (*n* = 3). These disks were placed in 1 mL dimethylformamide (DMF) for chlorophyll and carotenoid extraction and the absorbance spectra were recorded using a spectrophotometer (U-2000; Hitachi, Japan). Total chlorophyll and carotenoid levels were calculated using absorbance at 470, 647, and 663 nm according to Lichtenthaler (1987).

### RNA Extraction and Quantitative RT-PCR

Tuber tissues were cored using a cork borer (8 mm diameter) and six disks of 5 mm thickness including the skin and periderm were harvested from each tuber as technical replicates and were frozen in liquid nitrogen under safe green light. One tuber was treated as one biological replicate and there were three biological replicates for each data point (*n* = 3). Freeze dried disks were ground and homogenized into powder. RNA was extracted from 0.5 g of powdered tissue as described previously (Ducreux et al., 2008). First-strand cDNA templates were generated by reverse transcription using an oligo-d(T) primer (Primerdesign Ltd., Southampton, United Kingdom) according to the manufacturer’s instructions. PrecisionPLUS qPCR Master Mix (Primerdesign Ltd.) containing SYBR^®^Green was used to amplify the desired PCR product from each cDNA sample in the following program: denaturation of the template at 95°C for 15 min, followed by 40 cycles of: melting at 95°C for 10 s; annealing and extension at 60°C for 20 s. Relative expression values were calculated using the 2^−ΔΔ*CT*^ method (Livak and Schmittgen, 2001) using *TUBB1* encoding tubulin β-1 chain protein as an internal control (Taylor et al., 1994). All primer sets used are given in Supplementary Table S7.

### Microarray Analysis

Tuber tissues were cored using a cork borer (8 mm diameter) and six disks of 5 mm thickness including the skin and periderm were harvested from each tuber as technical replicates and were frozen in liquid nitrogen under safe green light. One tuber was treated as one biological replicate and from this an RNA sample was extracted for probing one array slide. There were three biological replicates in each treatment (*n* = 3). A custom Agilent microarray was used throughout, designed to the predicted transcripts from the potato (DM) genome as described (Hancock et al., 2014). The experimental design and datasets are available at ArrayExpress^1^ (accession E-MTAB-7707). A single-channel microarray design was utilized with all tuber RNA samples labeled with Cy3 dye. A total of 21 microarrays were processed, consisting of three biological replicates for each tuber sample (WL 6 h; B 6 h; R 6 h; WL 24 h; B 24 h; R 24 h; Dark). RNA labeling and downstream microarray processing was performed as described (Morris et al., 2014). Feature extraction (Agilent FE v.12.03.02) datasets for each array were loaded as single-channel data into GeneSpring (v.7.3) software for further analysis. Data were normalized using default single-channel settings: intensity values were set to a minimum of 0.01 and data from each array were normalized to the 50th percentile of all measurements on the array. Unreliable data, flagged as absent in all replicate samples by the FE software, were discarded. Statistical filtering of data to identify differentially expressed transcripts between light treatments (Dark v WL; Dark v R; Dark v B) at each time point (6 h; 24 h) was made using independent volcano plots. Thresholds of greater than 2-fold change and a Student’s *t*-test value of *P* ≤ 0.01 was applied. Heatmaps were created from selected genelists with specific known annotations using Pearson correlation with average linkage. Venn diagrams were created using the web-interactive bioinformatics site (Heberle et al., 2015).

Gene lists were analyzed for gene ontology (GO) term enrichment using the AgriGO v2 package^2^ (Tian et al., 2017). The Phureja DM1-3 PGSC gene ID for the entire genome was used as background, and Singular Enrichment Analysis performed using the Fisher test method, Yekuteli FDR, at a *P*-value ≤ 0.05 with a minimum of 10 mapping entries.

### Glycoalkaloid Extraction

The freeze dried and ground powdered samples used for RNA extraction and gene expression analysis were also utilized for GA analysis. As above, one tuber was treated as one biological replicate and there were three biological replicates in each data point (*n* = 3). Homogenized powdered material (100 mg) was extracted in 2 mL of 1:1 HPLC grade methanol:water (J.T Baker, United Kingdom) acidified with 0.1 % (v/v) mass spectrometry grade formic acid (P/N A117-50, Fisher Scientific, United Kingdom) and spiked with internal standard (1 μM reserpine: PN 83580, crystallized, ≥ 99.0% (HPLC), Sigma Aldrich, United Kingdom). The samples were vortex mixed for 20 s and shaken at maximum speed on a Heidolph Multi Reax shaker platform for 1 h. The samples were then centrifuged at 4075 × *g* for 10 min, the supernatant filtered (0.45 μm PTFE filter) and transferred to a micro-vial and capped (PN 60180-507 and 60180-516, Thermo-Fisher Scientific, United Kingdom).

### UHPLC-MS Analysis

UHPLC separations were performed with an Agilent 1260 Infinity HPLC system coupled with a 6230 TOF/MS system operated under Agilent Mass Hunter software (Agilent, Cheadle, United Kingdom). The potato extracts were subjected to LC-MS analysis using a high throughput 22 min UHPLC separation. In addition to the experimental sample set, reference, quality control and blank samples were analyzed to provide high levels of data quality assurance. A ten point calibration curve for α-chaconine and α-solanine was also obtained for the following serial dilution series: 250 > 125 > 62.5 > 31.25 > 15.625 > 7.8125 > 3.9063 > 1.9531 > 0.9766 > 0.4883 μM. A full description of the method is available within the Supplementary Data (Supplementary Data Sheet S1).

### UHPLC-MS Data Processing and Quantification

The LC-MS raw data profiles were imported into the Agilent MassHunter TOF Quantitation software module and peak areas integrated applying the Agile 1 method for each detected GA, inclusive of: solasonine, solanine malonyl, α-solanine, α-chaconine, chaconine malonyl, and an unknown GA of *m/z* 852.57 at RT of 10.8 min. QC and reference samples were applied to define data quality, only peak areas that showed less than 10% RSD across all QC and reference samples were taken forward to data analysis. The integrated peak areas for each sample and glycoalkaloid were relatively quantified against the calibration curve for either α-chaconine or α-solanine. Sample chromatographs with the retention times of the GA peaks are also presented in Supplementary Data Sheet S1.

## Results

### Tuber Chlorophyll and Carotenoid Accumulation Is Dependent on Light Intensity and Duration

**FIGURE 1:**
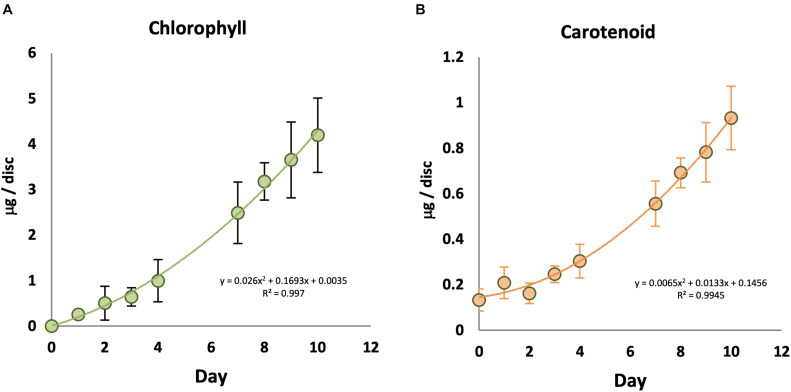
Time course of chlorophyll and carotenoid accumulation in KE tubers exposed to white light. Tubers were exposed to 14 μmol/m^2^/s white light at 18°C for an increasing number of days and total chlorophyll **(A)** and carotenoid **(B)** accumulation was measured. Data shown are mean ± S.D. (*n* ≥ 3 independent biological replicates).

In order to understand the relationship between light and chlorophyll accumulation, KE tubers were initially given a series of low to high intensities of WL for 7 days (Supplementary Figure S1A). No chlorophyll was observed in the absence of light, and total chlorophyll levels in the tubers increased with increasing light intensity and saturated at or below 14 μmol/m^2^/s at 18°C. Carotenoid accumulation was also detected in a light intensity dependent manner, but appeared to saturate at slightly lower fluences than chlorophyll at the 7 day time point. Using this saturating fluence rate, we followed a time course for chlorophyll and carotenoid synthesis in KE tubers under WL (Figure 1). Again, there was no detectable chlorophyll in the KE tubers on day 0 prior to the light treatment (Figure 1A). Under our assay conditions, chlorophyll accumulation was detected in tubers irradiated with WL for more than two days and chlorophyll levels increased with increasing periods of light exposure (Figure 1A; Supplementary Figure S1B). In contrast to the situation for chlorophyll, there was a background level of carotenoids detected even in tubers that had received no light treatment on day 0 (Figure 1B), consistent with the result on day 7 in the absence of light (Supplementary Figure S1A). A steady increase in carotenoid accumulation was detected in tubers exposed to light with an increase observed after three days. The data also showed that the increase in chlorophyll and carotenoid accumulation in KE tubers was approximately linear after an initial lag phase.

### Chlorophyll and Carotenoid Accumulation Is Induced by B and R Light

**FIGURE 2:**
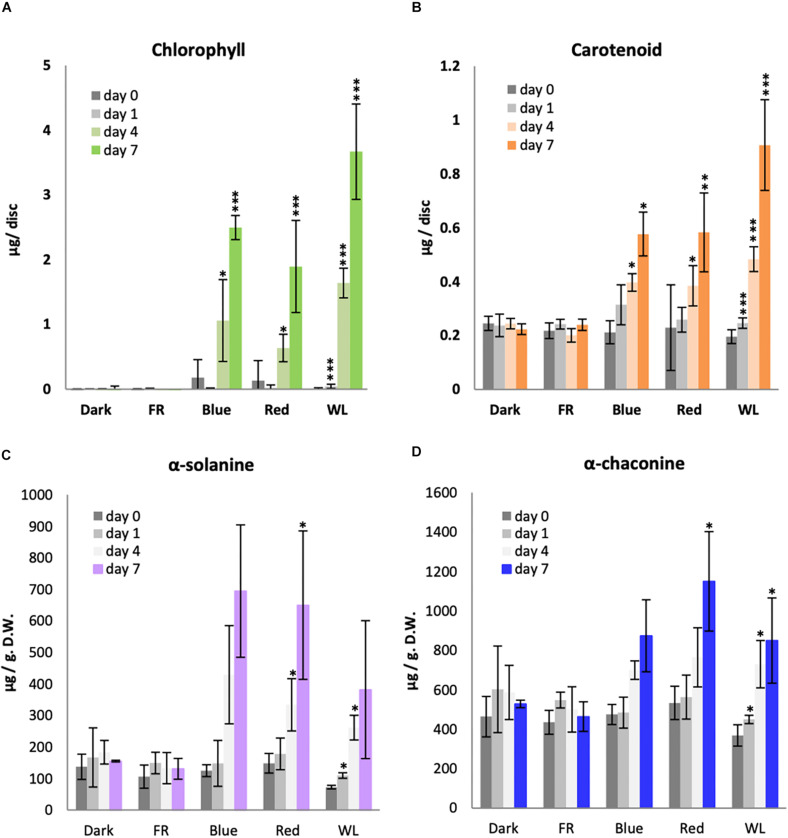
Light induction of chlorophyll, carotenoid and glycoalkaloid accumulation in KE tubers. Tubers were exposed to far-red (FR), Blue, Red, white light (WL) or kept in darkness (Dark) at 18°C for 1, 4, or 7 days and the accumulation of total chlorophyll **(A)**, carotenoids **(B)** or the GAs, α-solanine **(C)** and α-chaconine **(D)** was measured. Data shown are mean ± S.D. (*n* = 3 independent biological replicates) except for the GA measurements of tubers exposed to Blue, FR, and Dark where the mean of 2 independent biological replicates is shown. Asterisks indicate statistical differences between day 0 and respective treatments as determined by Student’s *t*-test (*p* < 0.05*, *p* < 0.001**, *p* < 0.0001***). Note the statistical analysis was performed only for those data with 3 or more independent biological replicates.

Plants have two major classes of photoreceptors known to regulate both chlorophyll and carotenoid accumulation: B light-responsive cryptochromes and R and FR light-responsive phytochromes. We therefore investigated the effectiveness of B, R and FR light in comparison with WL for the induction of chlorophyll and carotenoids in KE tubers over a 7 day period. As seen previously, there was no detectable chlorophyll present in the tubers kept in darkness for 7 days (Figure 2A). Similarly, tubers exposed to FR did not accumulate a detectable level of chlorophyll. This was also expected as the chlorophyll synthesis enzyme protochlorophyllide oxidoreductase has a strict light dependency that is not supported by wavelengths in the FR region (Heyes and Hunter, 2005). There was a background level of carotenoids detected in tubers irrespective of whether they were kept in darkness or were exposed to FR light and this did not significantly change (*p*-values > 0.05) over the 7 day time course (Figure 2B). A steady increase of both chlorophyll and carotenoid accumulation was detected in tubers exposed to B and R light, with the highest levels detected on day 7 (Figure 2). In both cases, more pigment accumulation was observed in WL than under monochromatic light sources, probably because the total intensity was higher for WL (14.5 μmol/m^2^/ sec) compared to that of B light (3.5 μmol/m^2^/ sec) and R (7.5 μmol/m^2^/ sec). The data in Figure 2 are presented on a per area basis, however the results for chlorophyll were identical if the data were presented with respect to dry weight (Supplementary Figure S2A). In addition, no differences were seen comparing accumulation of chlorophyll *a* and *b* (Supplementary Figure S2B).

### Chlorophyll and Carotenoid Biosynthesis Genes Are Induced by WL, B, and R, but Not by FR Light

**FIGURE 3:**
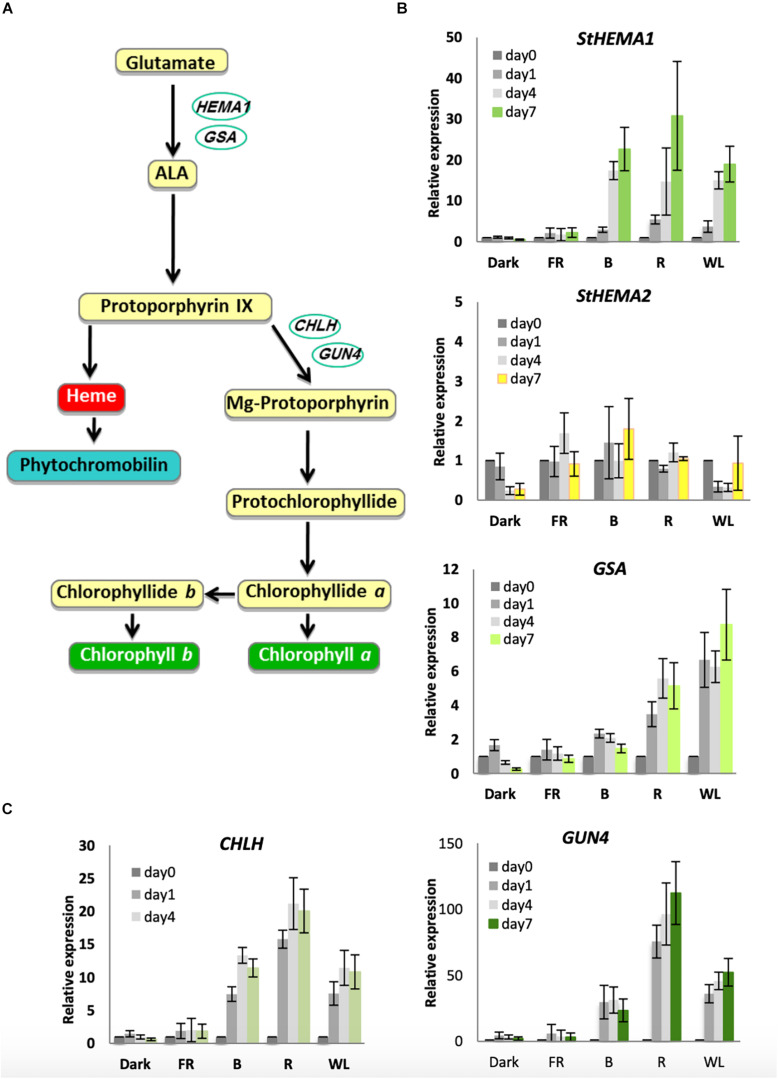
Light induction of genes encoding chlorophyll biosynthesis enzymes in KE tubers. **(A)** The tetrapyrrole biosynthesis pathway with relevant genes indicated. *HEMA1* and *GSA* encode the enzymes, glutamyl-tRNA reductase and glutamate-1-semialdehyde-2,1-aminomutase, respectively, and are involved in the conversion of glutamate to 5-aminolevulinic acid (ALA), the rate-limiting step in tetrapyrrole synthesis. *CHLH* encodes a subunit of Mg-chelatase that commits tetrapyrroles to chlorophyll synthesis and *GUN4* is a regulator of this enzyme. **(B,C)** Tubers were exposed to far-red (FR), blue (B), red (R), or white light (WL) or kept in darkness (Dark) at 18°C for 0, 1, 4, or 7 days and RNA was extracted. Expression was determined by quantitative RT-PCR and is shown relative to Dark 0 days and normalized to *β-TUBULIN*. Data shown are mean ± S.D. (*n* = 3 independent biological replicates).

It has been shown previously in Arabidopsis that chlorophyll biosynthesis genes are induced by cryptochrome and phytochrome photoreceptors upon exposure to B and R light, respectively (McCormac et al., 2001; McCormac and Terry, 2002; Stephenson and Terry, 2008). However, this has never been tested in potato tubers. The rate-limiting step of chlorophyll biosynthesis is catalyzed by GluTR (see Figure 3A for the chlorophyll biosynthesis pathway). In Arabidopsis this is encoded by two functional genes: *HEMA1*, which is light-regulated and responsible for photosynthetic pigment synthesis, and *HEMA2*, which is not light induced (Ilag et al., 1994; McCormac et al., 2001; Ujwal et al., 2002). Analysis of *HEMA* sequences in the potato genome sequence database annotated by the International Potato Genome Sequencing Consortium (PGSC) revealed three genes that were similar to Arabidopsis *HEMA1* and *HEMA2* with over 80% similarity to *HEMA1* at the amino acid level. The predicted amino acid sequence of all three genes contained the conserved NADPH- and ALA-binding consensus motifs (Zhao et al., 2014). Of the three genes identified, two transcripts, PGSC0003DMT400016326 and PGSC0003DMT400008034, encoded proteins with putative chloroplast localization signals at the *N*-terminus and are therefore likely to be functional in the chloroplast (Supplementary Figure S3). PGSC0003DMT400016326 is more similar to Arabidopsis *HEMA1* (Supplementary Figure S4A) and we have termed the associated gene St*HEMA1*. PGSC0003DMT400008034 does not show obvious similarity to Arabidopsis *HEMA1* or *HEMA2* and we have named this associated gene St*HEMA2.* We tested both genes, together with *GSA*, which encodes the next enzyme in the pathway, glutamate-1-semialdehyde 2,1-aminomutase, and has been shown in Arabidopsis to be moderately light regulated (McCormac and Terry, 2002). Potato tubers were treated with WL, B, R, and FR or kept in darkness for 0, 1, 4 or 7 days and RNA was isolated for quantitative RT-PCR analysis. The relative transcript level in each RNA sample was calculated using tubulin as an internal control (Taylor et al., 1994) with data plotted relative to expression on day 0 (Figure 3). As shown in Figure 3B, St*HEMA1* showed a strong induction in response to WL, B, and R (up to 30-fold), but no expression in the dark or after FR light. *GSA* also showed induction by light, but this was more moderate (up to 8-fold) with R and WL clearly more effective than B. This is in contrast to St*HEMA1*, for which B and R were as or more effective than WL (Figure 3B). Interestingly, neither St*HEMA1* nor *GSA* showed induction under FR light. In contrast to these two genes, St*HEMA2* showed no light induction and therefore behaved more, similarly, to Arabidopsis *HEMA2* (Ujwal et al., 2002).

The next major regulatory step in the chlorophyll biosynthesis pathway is the insertion of Mg^2+^ by magnesium chelatase (Figure 3A). In Arabidopsis, the *CHLH* gene was shown to be far more light responsive than *CHLD* or the two *CHLI* genes (Stephenson and Terry, 2008). We therefore examined *CHLH* expression together with the regulator *GUN4* that also shows a strong response to light in Arabidopsis (Stephenson and Terry, 2008). Both genes showed strong induction under WL, B, and R, with R light considerably more effective than WL or B with over 100-fold induction of *GUN4* observed (Figure 3C). Again, in contrast to what has been observed in Arabidopsis, no induction was seen for either gene under FR light (Stephenson and Terry, 2008). Together these results suggest that both cryptochromes and phytochromes are the likely photoreceptors that regulate chlorophyll accumulation in KE tubers.

**FIGURE 4:**
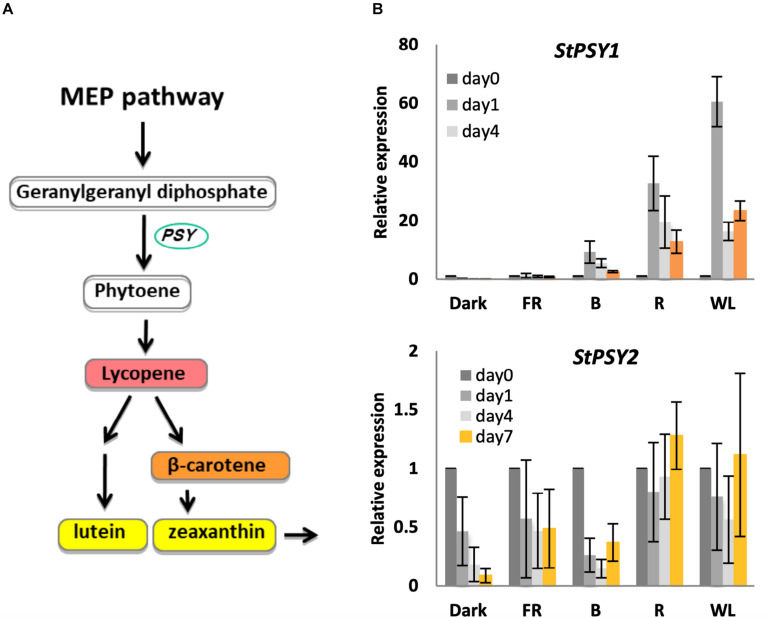
Light induction of phytoene synthase gene expression in KE tubers. **(A)** The carotenoid biosynthesis pathway with *PSY* genes encoding phytoene synthase indicated. **(B)** Tubers were exposed to far-red (FR), blue (B), red (R), or white light (WL) or kept in darkness (Dark) at 18°C for 0, 1, 4, or 7 days and RNA was extracted. Expression of *PSY* genes was determined by quantitative RT-PCR and is shown relative to Dark 0 days and normalized to *β-TUBULIN*. Data shown are mean ± S.D. (*n* = 3 independent biological replicates).

The first committed step in carotenoid biosynthesis is catalyzed by phytoene synthase (encoded by *PSY*), which converts geranylgeranyl diphosphate to phytoene (Pasare et al., 2013; Figure 4). The potato genome encodes at least two *PSY* genes, PGSC0003DMT400061846 (*PSY1*), and PGSC0003DMT400043103 (*PSY2*), with 82 and 75 % similarity to Arabidopsis *PSY* (At5g17230), respectively (Supplementary Figure S5). We therefore tested whether changes in these *PSY* genes could account for the observed changes in carotenoid levels. As shown in Figure 4, *PSY1* showed a very strong induction (up to 60-fold) in R and WL with expression peaking at day 1 in contrast to the chlorophyll synthesis genes. In addition, some induction was seen by B light, but none in FR light or in tubers kept in darkness. In contrast, *PSY2* showed no induction by light (Figure 4).

### Glycoalkaloid Synthesis Is Induced by WL, B, and R, but Not by FR Light

In order to test if GA accumulation is mediated by B, R, or FR light, KE tuber tissues harvested for chlorophyll and carotenoid analyses under these light conditions were also tested for GA accumulation.

Of all GA species, α-solanine and α-chaconine are the most abundant in potato tubers and account for more than 90 % of the total GAs (Griffiths et al., 1997; Sotelo and Serrano, 2000). KE is a variety known to accumulate relatively low levels of GAs (Valcarcel et al., 2014), however, some GA accumulation was still observed in the tubers before exposure to light (Figures 2C,D). Both α-solanine and α-chaconine accumulated over time under WL, B, and R, with induction seen by day 4 and the highest levels detected after 7 days. Indeed, B and R light alone generally appeared to be more effective than WL, in contrast to the situation for chlorophyll or carotenoid synthesis. Neither α-solanine nor α-chaconine accumulated in tubers exposed to FR light or kept in darkness.

### Glycoalkaloid Biosynthesis Genes Are Induced by WL, B, and R, but Not by FR Light

**FIGURE 5:**
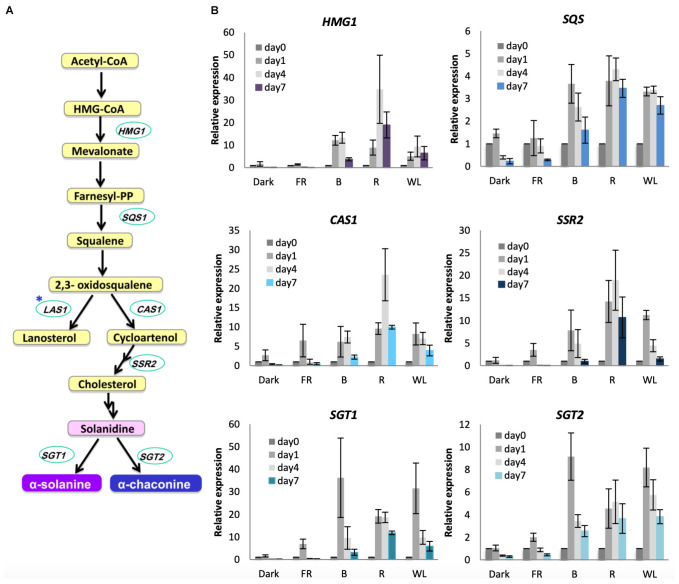
Light induction of genes encoding glycoalkaloid biosynthesis enzymes in KE tubers. **(A)** The GA biosynthesis pathway with relevant genes indicated. *HMG1, SQS, CAS1, SSR2 SGT1* and *SGT2* encode the enzymes 3-hydroxy-3-methylglutaryl coenzyme A reductase, squalene synthase, cycloartenol synthase 1, sterol side chain reductase 2, solanidine galactosyltransferase1 and solanidine glucosyltransferase 2, respectively. Asterisk indicates the gene encoding lanosterol synthase 1 (*LAS1*) which leads to the branch pathway for cholesterol biosynthesis. **(B)** Tubers were exposed to far-red (FR), blue (B), red (R), or white light (WL) or kept in darkness (Dark) at 18°C for 1, 4, or 7 days and RNA was extracted. Expression was determined by quantitative RT-PCR and is shown relative to Dark 0 days and normalized to *β-TUBULIN*. Data shown are mean ± S.D. (*n* = 3 independent biological replicates).

To understand the molecular basis of the increased GA synthesis we tested the induction of GA biosynthesis genes under WL, B, R, and FR. GAs in *S. tuberosum* are synthesized from the precursor acetyl-CoA (Figure 5A). 3-hydroxy-3-methyl-glutaryl-coenzyme A (HMG-CoA) reductase (encoded by *HMG1*), which catalyzes the reduction of HMG-CoA to yield mevalonate is the rate-limiting step in this pathway (Burg and Espenshade, 2011). Squalene synthase (*SQS*) also functions in the main branch of the pathway. The first branchpoint from 2,3-oxidosqualene either leads to the formation of cycloartenol synthesized by cycloartenol synthase (*CAS1*) or lanosterol via *LAS1*. The cycloartenol branch leads to the formation of cholesterol via sterol side chain reductase (*SSR2*), and subsequently the GAs α-solanine and α-chaconine are synthesized by solanindine galactosyltransferase (*SGT1*) and solanidine glucosyltransferase (*SGT2*) from solanidine. We tested these seven genes for their response to light over 7 days (Figure 5B, Supplementary Figure S6). The genes *HMG1*, *SQS*, *CAS1*, *SSR2*, *SGT1* and *SGT2* were induced by WL, B, and R, although there was variation in the extent of induction and the relative contribution of the different light wavelengths (Figure 5B). *SGT1* in particular showed a very rapid and strong induction (about 30-fold) in WL and B after just 1 day, with *SGT2* also showing strongest expression after 1 day of light treatment (Figure 5B). All of these six genes showed little or no increase in expression by FR light or in darkness. In contrast to these genes, *LAS1*, that is not required for GA production, showed little change in expression after any light treatment (Supplementary Figure S6A) and *SSR1/DWF1*, which encodes a reductase leading to the steroid biosynthesis branch of the pathway, also showed little change (Supplementary Figure S6B). These results show that there is a specific induction of genes leading to α-solanine and α-chaconine synthesis and that gene expression is activated throughout the GA biosynthesis pathway.

### Comparative Analysis of the Tuber Transcriptome After WL, B, or R Exposure

In order to obtain an insight into how light is affecting changes in gene expression across the potato transcriptome, we performed transcriptomic analyses using the solAarray^3^ microarray. Our quantitative RT-PCR analyses demonstrated an increase in accumulation of transcripts encoding enzymes of the key steps of chlorophyll, carotenoid, and GA biosynthesis by 24 h post light exposure and we therefore selected 24 h as one time point to be analyzed. However, we also wanted to gain an understanding of the earliest changes in transcriptome expression following light exposure and also included a 6 hour time point in our analysis. We predicted that this earlier point may allow us to identify important transcription factors mediating light-induced changes in the transcriptome.

**FIGURE 6:**
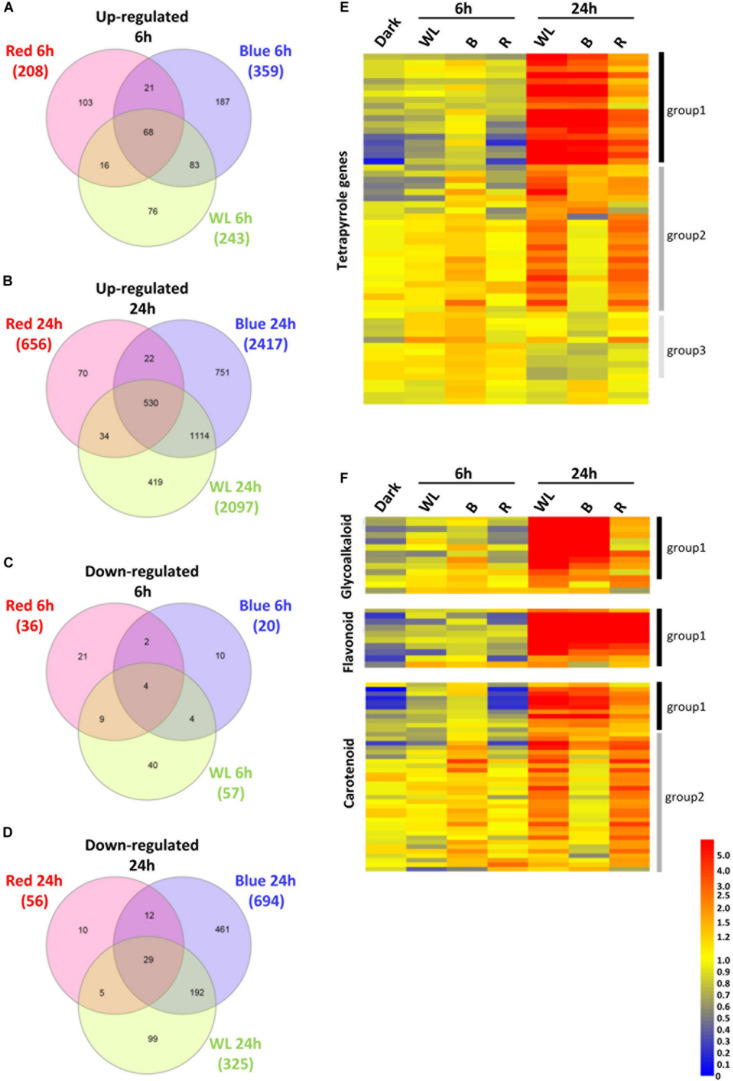
Transcriptome analysis of KE tubers exposed to red, blue or white light. **(A–D)** Venn diagrams showing genes that are more than 2-fold statistically significantly up-regulated after 6 h **(A)**, or 24 h **(B)** and down-regulated after 6 h **(C)**, or 24 h **(D)** exposure to red, blue, or white light (WL). Numbers in brackets indicate the number of genes that were found regulated by the given light condition and genes are listed in Supplementary Tables S1–S6. **(E,F)** Heat maps showing the normalized expression levels of selected genes that are known to be involved in the tetrapyrrole **(E)**, GA, flavonoid, and carotenoid **(F)** biosynthesis pathways. The genes shown are listed in Supplementary Table S8 **(E)** and Supplementary Tables S9–S11 **(F)**. Heat maps were created using GeneSpring ver. 7.3 and hierarchical clustering analysis was performed using Pearson correlation. Defined expression profiles corresponding to Group 1 (solid black vertical lines), Group 2 (dark gray) and Group 3 (light gray) are indicated. See text for details.

Tubers were exposed to WL, B, or R 6, 24 h or kept in darkness, and the changes in gene expression between darkness and each light treatment were determined. The expression of a total of 300, 379, and 244 genes were found to be statistically significantly different (*p*-values < 0.01) by > 2-fold after 6h exposure to WL, B, or R, respectively (Supplementary Tables S1–S3). Of these genes, 243, 359, and 208 genes were more than two-fold up (Figure 6A) while 57, 20 and 36 were down-regulated (Figure 6C) by WL, B and or R light, respectively. Compared to the 6 h time point, a relatively large number of genes were differentially regulated by 24 h exposure to light. A total of 2422, 3111, and 712 genes were found to be statistically significantly different (*p*-values < 0.01) by > 2-fold after 24h exposure to WL, B, or R light, respectively (Supplementary Tables S4–S6). Of these genes, 2097, 2417, and 656 genes were up-regulated (Figure 6B) while 325, 694 and 56 were down-regulated (Figure 6D) by WL, B and or R light, respectively.

To obtain a general overview of the classes of gene expression changes on exposure to light, we performed gene ontology (GO) enrichment analysis on genes that were up-regulated by WL at 6 h and 24 h using the AgriGO v2 platform (Tian et al., 2017; see section “MATERIALS AND METHODS”). In general, increases were observed in a broad range of metabolic processes and in transcription with the most notable induction being in photosynthesis associated genes (Supplementary Figure S9).

**FIGURE 7:**
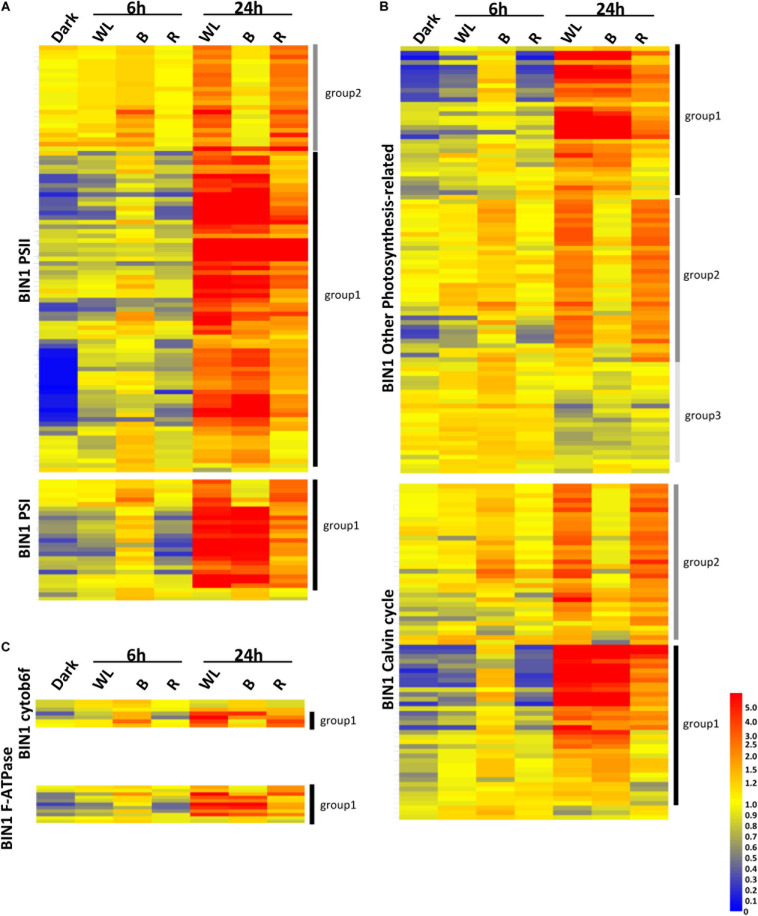
Transcriptome analysis of photosynthetic genes in tubers exposed to red, blue, or white light for 0, 6, and 24 h. **(A–C)** Heat maps showing the normalized expression levels of the MapMan BIN1 genes encoding PSII and PSI proteins **(A)**, cytob6f and F-ATPase proteins **(C)** or Calvin cycle and other photosynthesis related proteins **(B)**. The genes shown are listed in Supplementary Tables S12–S16. Heat maps were created using GeneSpring ver. 7.3 and hierarchical clustering analysis was performed using Pearson correlation. Defined expression profiles corresponding to Group 1 (solid black vertical lines), Group 2 (dark gray) and Group 3 (light gray) are indicated. See text for details.

In the KE tuber response to 24 h exposure of light, 530 (Figure 6B) and 29 (Figure 6D) genes were more than two-fold up- and down-regulated, respectively, in all three light conditions. The 530 genes that were significantly up-regulated included carotenoid and chlorophyll biosynthesis genes such as *PSY*, *HEMA1*, *CHLH*, and *GUN4* (Supplementary Tables S4–S6) that we had previously shown to be induced using qPCR (Figures 3, 4). We next analyzed all of the genes annotated as being involved in the synthesis of chlorophyll (Figure 6E), GAs, flavonoids, and carotenoids (Figure 6F) and created expression heat maps of these gene sets (see Supplementary Tables S8, S9 for gene lists used). All of the genes in these categories were up-regulated in all three light conditions and the transcript levels were generally higher after 24 h light exposure than after 6 h. In addition, photosynthesis-related genes (MapMan BIN1; Figures 7A,B) were also mostly induced over 24 h in all three light conditions. These results were consistent with our qPCR analysis.

One noticeable feature identified in the gene expression heat maps is that light regulation of genes falls into three distinct groups. Firstly, for one group, gene expression level is elevated at both 6 and 24 h and in most cases this happens progressively so that the level of expression is higher at 24 h compared to that at 6 h (Figures 6, 7). We have called this set of genes Group 1. The second distinct group of genes, Group 2, are genes that show induced expression under WL, B, and R at 6h, but in contrast to group 1 there is a reduced level of transcripts in B after 24 h compared to R and WL, which retain progressive upregulation of gene expression. The third group of genes, Group 3, showed a relatively moderate level of expression at 6 h with no induction apparent after 24 h under all light conditions. This group was less commonly observed and only for tetrapyrrole biosynthesis (Figure 6E) or photosynthesis-related (Figure 7B) genes. The Group 3 tetrapyrrole genes are those that would be predicted to encode proteins required for extra-plastidic supply of heme (Mochizuki et al., 2010). In general, tetrapyrrole and carotenoid biosynthesis genes and most major categories of photosynthesis genes showed a mixture of Group 1 and Group 2 induction profiles. In contrast, flavonoid and glycoalkaloid biosynthesis genes showed a predominantly Group 1 induction profile. It is likely that each group represents the output from distinct signaling pathways.

To further investigate these signaling pathways and to determine whether there is similarity between genes involved in this response in the potato tuber and young seedlings, we examined the expression profile of transcription factors (TFs) contained within MapMan BIN27.3 (Thimm et al., 2004). Of the 2918 annotated TFs, 1732 genes were more than 2-fold upregulated in at least one of the 6 light conditions when compared to the dark control (Supplementary Table S17) and 18 TFs were consistently and progressively induced at both 6 and 24 h and under all three light qualities (Table 1). Amongst these genes, the homologues of Arabidopsis TFs of the b-zip type, *HY5*, pseudo-response regulators, *PRR2*, *PRR5* and *PRR9* and the transcripts of several *AP2/EREBP* transcription factors were found enriched under all light conditions. In addition, transcripts of seven TF genes showed more than 2-fold induction at 6 h compared to the dark control with expression reduced to less than the dark control at 24 h (Table 2). Such a transient upregulation has been linked to an early role in light signaling (Tepperman et al., 2001) and indeed this group contained several *Dof* zinc finger type TFs that have been implicated in the regulation of light signaling. In addition, 236 TF genes were more than 2 fold down-regulated in at least one of the light conditions (Supplementary Table S18). Amongst these genes were ethylene response regulators and the jasmonate repressor, *JAZ3*.

**TABLE 1:** Transcription factors showing more than 2 fold up regulation at 6 and 24 h in tubers exposed to blue, red, and white light.

UniRef based putative functional annotation	Primary Accession	MapMan bin	MapMan name
Pseudo-response regulator 9	PGSC0003DMT400029402	27.3.66	RNA.regulation of transcription.Psudo ARR transcription factor family
Transcription factor HY5	PGSC0003DMT400053573	27.3.35	RNA.regulation of transcription.bZIP transcription factor family
COL domain class transcription factor	PGSC0003DMT400068177	27.3.7	RNA.regulation of transcription.C2C2(Zn) CO-like, Constans-like zinc finger family
Zinc finger B-box protein	PGSC0003DMT400069497	27.3.7	RNA.regulation of transcription.C2C2(Zn) CO-like, Constans-like zinc finger family
Pseudo response regulator	PGSC0003DMT400062232	27.3.5	RNA.regulation of transcription.ARR
Heat shock factor	PGSC0003DMT400082751	27.3.23	RNA.regulation of transcription.HSF,Heat-shock transcription factor family
APETALA2	PGSC0003DMT400016584	27.3.3	RNA.regulation of transcription.AP2/EREBP, APETALA2/Ethylene-responsive element binding protein family
Pseudo-response regulator 5	PGSC0003DMT400001573	27.3.66	RNA.regulation of transcription.Psudo ARR transcription factor family
APETALA2	PGSC0003DMT400016585	27.3.3	RNA.regulation of transcription.AP2/EREBP, APETALA2/Ethylene-responsive element binding protein family
Fructokinase 2	PGSC0003DMT400069498	27.3.7	RNA.regulation of transcription.C2C2(Zn) CO-like, Constans-like zinc finger family
TAF-3	PGSC0003DMT400084206	27.3.35	RNA.regulation of transcription.bZIP transcription factor family
Apetala 2	PGSC0003DMT400065313	27.3.3	RNA.regulation of transcription.AP2/EREBP, APETALA2/Ethylene-responsive element binding protein family
Apetala 2	PGSC0003DMT400065312	27.3.3	RNA.regulation of transcription.AP2/EREBP, APETALA2/Ethylene-responsive element binding protein family
RAV transcription factor	PGSC0003DMT400002125	27.3.3	RNA.regulation of transcription.AP2/EREBP, APETALA2/Ethylene-responsive element binding protein family
Auxin response factor 16	PGSC0003DMT400062489	27.3.4	RNA.regulation of transcription.ARF, Auxin Response Factor family
DNA binding protein	PGSC0003DMT400028368	27.3.50	RNA.regulation of transcription.General Transcription
F-box family protein	PGSC0003DMT400046598	27.3.8	RNA.regulation of transcription.C2C2(Zn) DOF zinc finger family
Putative calcium-dependent protein kinase CPK1 adapter protein 2	PGSC0003DMT400009348	27.3.51	RNA.regulation of transcription.General Transcription, TBP-binding protein

*Eighteen genes encoding transcription factors of BIN27.3 showed progressive induction of more than two fold in tubers exposed to WL, B, or R for 6 and 24 h.*

**TABLE 2:** Transcription factors showing more than 2 fold up regulation at 6 h and down regulation at 24 h in tubers exposed to blue, red and white light.

UniRef based putative functional annotation	Primary Accession	MapMan bin	MapMan name
Transcription factor B-box type	PGSC0003DMT400067337	27.3.7	RNA.regulation of transcription.C2C2(Zn) CO-like, Constans-like zinc finger family
LOL1 (LSD ONE LIKE 1)	PGSC0003DMT400045143	27.3.99	RNA.regulation of transcription.unclassified
TAF-3	PGSC0003DMT400084204	27.3.35	RNA.regulation of transcription.bZIP transcription factor family
Nuclear transcription factor Y subunit A-4	PGSC0003DMT400054418	27.3.14	RNA.regulation of transcription.CCAAT box binding factor family, HAP2
Dof zinc finger protein	PGSC0003DMT400050273	27.3.8	RNA.regulation of transcription.C2C2(Zn) DOF zinc finger family
MYB transcription factor	PGSC0003DMT400028709	27.3.26	RNA.regulation of transcription.MYB-related transcription factor family
Dof zinc finger protein	PGSC0003DMT400050272	27.3.8	RNA.regulation of transcription.C2C2(Zn) DOF zinc finger family

*Seven genes encoding transcription factors of BIN27.3 showed more than two fold induction in tubers exposed to WL, B, or R for 6 h. This group of genes showed a lower level of expression at 24 h.*

### Chlorophyll and GA Accumulation by WL Is Alleviated by Supplementary FR Light

**FIGURE 8:**
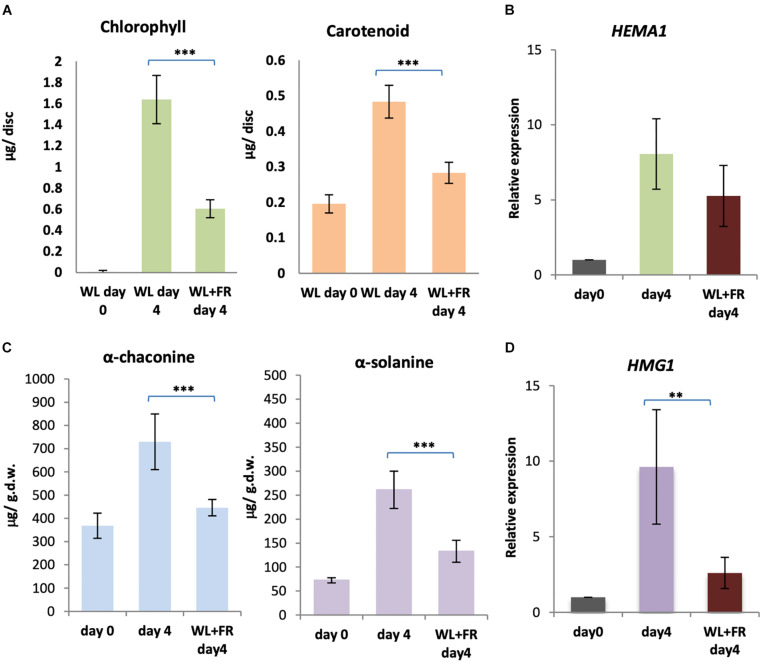
Far-red light alleviates the accumulation of chlorophyll and GA in white light. **(A–D)** Tubers were exposed to white light (WL) or white and far-red (FR) light for 4 days and the levels of chlorophyll and carotenoids **(A)** or α-solanine and α-chaconine **(C)** were measured. Expression of *HEMA1* **(B)** and *HMG1* **(D)**, encoding the rate-limiting steps of the chlorophyll and GA biosynthesis pathways, respectively, was determined by quantitative RT-PCR and is shown relative to Dark 0 days and normalized to *β-TUBULIN*. Data shown are mean ± S.D. (*n* = 3 independent biological replicates). Asterisks indicates statistical differences between the two treatments indicated by the line above as determined by Student’s *t*-test (*p* < 0.001**, 0.0001***).

Our results suggest that both cryptochromes and phytochromes are likely mediating chlorophyll and GA accumulation in light. However, there was no change in expression of biosynthetic enzyme genes under FR light, suggesting the absence of a FR high irradiance response mediated by phytochrome A in the control of this process (Mancinelli and Rabino, 1978; Hennig et al., 2000). Therefore, we reasoned that supplementary FR may reduce the amount of the active, FR absorbing Pfr form of phytochrome and thereby reduce both greening and GA accumulation. In order to test this, KE tubers were exposed to either WL only or WL with supplementary FR light for four days and the levels of chlorophyll, carotenoids, α-solanine and α-chaconine determined. Both chlorophyll (*p* < 0.0001) and carotenoid (*p* < 0.0008) levels were significantly reduced in KE tubers exposed to WL in the presence of FR light compared to exposure to WL alone for four days (Figure 8A). Similarly, α-solanine (*p* < 0.0008) and α-chaconine (*p* < 0.0004) levels were also significantly reduced in KE tubers in supplementary FR light (Figure 8C). In all cases inhibition by FR was incomplete as expected considering the contribution of B light, but nevertheless, was very effective with an approximately 60–80% reduction observed. This confirms the major role of phytochromes in mediating light induction of greening and GA synthesis. We next tested whether these changes were reflected in changes in gene expression. The two genes, *HEMA1* and *HMG1* encoding the rate-limiting enzymes for chlorophyll and GA biosynthesis, respectively, were examined and showed lower expression with supplementary FR light (Figures 8B,D), although this was only significant at the 5% level for *HMG1* (Figure 8D). A similar trend was seen for *CHLH* and *CAS1*, but not for other genes tested (Supplementary Figure S7).

## Discussion

It has long been accepted that light induces greening and GA accumulation in potato tubers. The intensity, duration, and spectral characteristics of light in induction of tuber greening have been studied in many cultivars over the last 70 years (Virgin and Sundqvist, 1992; Percival, 1999; Tanios et al., 2018). However, the mechanisms underlying the light induction of both chlorophyll and GA accumulation are still not well understood. Here, we have used a popular commercial cultivar of *S. tuberosum*, King Edward, and analyzed the accumulation of chlorophyll, carotenoids, and GAs, as well as the induction of genes encoding key enzymes in chlorophyll and GA biosynthesis by monochromatic B, R, and FR light as well as WL. Chlorophylls *a* and *b*, and the GAs, α-solanine and α-chaconine, have previously been shown to be induced by B and R light in *S. tuberosum* (c.v. Sebago) (Petermann and Morris, 1985). As expected, our results using monochromatic light sources with narrow peak wavelengths clearly indicate that B and R wavelengths of light induce chlorophyll, carotenoid and GAs in c.v. King Edward tubers (Figure 2) and support a role for cryptochromes and phytochromes in mediating the induction of chlorophyll and GA biosynthesis. Furthermore, our detailed gene expression analyses showed that genes encoding enzymes for the tetrapyrrole, phytoene, and GA biosynthesis are highly up-regulated in tubers after irradiation by WL, B, and R (Figures 3–6). Notably, neither expression of the biosynthesis genes nor accumulation of chlorophyll, carotenoid and GAs was observed in tubers exposed to FR light (Figures 2–5).

### Light Regulation of Chlorophyll, Carotenoid and GA Synthesis in Tubers

In order to see if the key regulatory steps of chlorophyll biosynthesis in tubers are controlled by light, we used qPCR to measure changes in gene expression. The results indicated that the genes *StHEMA1*, *GSA*, *CHLH*, and *GUN4* are all up-regulated by WL, B, and R providing a molecular basis for the observed accumulation of chlorophyll under these conditions. All land plant species investigated so far have at least two *HEMA* genes, one light regulated that is proposed to provide photosynthetic tetrapyrroles and one that is not responsive to light that is suggested to provide extraplastidic tetrapyrroles (Mochizuki et al., 2010). The potato genome encodes at least three amino acid sequences that show more than 70% similarity to that of AtHEMA1 (Supplementary Figures S3, S4B). Amongst these three, StHEMA1 and 2 were found to have a predicted chloroplast localization signal whereas StHEMA3 lacked ∼90 amino acids at the amino-terminal end (Supplementary Figure S3). Phylogenetic analysis indicated that the amino acid sequence PGSC0003DMT400016326 (StHEMA1) clusters closely with AtHEMA1 suggesting that *StHEMA1* is likely to be homologous to *AtHEMA1* (Supplementary Figure S4A). However, while Arabidopsis *AtHEMA1* is induced by continuous FR light at the seedling stage (McCormac et al., 2001; McCormac and Terry, 2002), *StHEMA1* was not significantly induced by FR light in the potato tuber (Figure 3B). Phytochrome A has a number of established roles in the regulation of potato growth and development (Heyer et al., 1995; Yanovsky et al., 1998; Yanovsky et al., 2000), including regulation of sprout growth via continuous FR light consistent with a phytochrome A-mediated FR high irradiance response (HIR) (Heyer et al., 1995). Our qPCR data confirm the expression of *PHYA* in potato tubers in darkness (Supplementary Figure S8) and it may be that the pigment pathways are not regulated by a FR-HIR in tubers. It would be interesting to test whether these responses are present in other tissues. In the closely related species tomato it was observed that seedlings did undergo a FR block of greening response which is consistent with the induction of chlorophyll biosynthesis genes under continuous FR light (van Tuinen et al., 1995).

The response to continuous R light is likely to be mediated mostly by phytochrome B. Potato has a similar complement of phytochromes to tomato and has two phytochrome B genes (Supplementary Figure S4B). During de-etiolation in tomato phyB1 and phyB2 work redundantly to regulate chlorophyll biosynthesis under R light (Weller et al., 2000). However, in the tomato study phytochrome A also had a role in the response to R light (Weller et al., 2000) and it is possible that all three phytochromes contribute to greening of potato tubers under R and WL. The ability of FR to reduce the WL response is most likely due to inhibiting the R component of the WL response through an inhibition of the active Pfr form of these phytochromes.

As with chlorophyll biosynthesis genes such as *AtHEMA1*, phytoene synthase (PSY) is known to be light induced in most land plants including Arabidopsis and potato (Ducreux et al., 2005). The potato genome encodes at least two genes *StPSY1* and *StPSY2* and the sequences of these genes showed a predicted chloroplast localization signal at the amino-terminus (Supplementary Figure S5). Interestingly, *StPSY1* was significantly induced by WL, B, and R, whereas *StPSY2* was not (Figure 4). *StPSY1* is therefore more similar to *AtPSY1* and the functional significance of *StPSY2* is unknown. In rice there are three PSY genes, two under phytochrome control and a third regulated by stress (Welsch et al., 2008). In would be interesting to test whether *StPSY2* is also stress regulated.

As expected from the light-dependent accumulation of α-solanine and α-chaconine (Figure 2C,D), key genes encoding enzymes for their biosynthesis are up-regulated by WL, B, and R (Figure 5B). In contrast to this, two genes encoding enzymes for cholesterol biosynthesis, *LAS1* and *SSR1/DWF1*, are not induced by any of the light conditions. Similar to genes involved in tetrapyrrole and carotenoid biosynthesis, GA biosynthesis genes were not up-regulated by FR light (Figure 5B). This result was consistent with our observation that there was no significant increase in α-solanine and α-chaconine (Figure 2C,D) in FR light (*p* > 0.05). These data also indicate that GA biosynthesis genes are likely to be regulated by cryptochromes and phytochromes.

### Transcriptomic Changes in Tubers on Transfer to Light

In order to understand the impact of light on the wider transcriptome, tubers were exposed to WL, B, and R for 6 and 24 h and changes in gene expression were analyzed using the solArray microarray. The shorter time course was used for this analysis to build a more immediate picture of the changes occurring during the greening process. In agreement with the data from the qPCR experiments, the results indicate that the tetrapyrrole, GA, flavonoid, and carotenoid biosynthesis-related genes are also generally up-regulated by WL, B, and R over this shorter time period (Figure 6). Interestingly, light induction followed three distinct patterns, which we defined as Groups 1-3. Groups 1 and 2 contained the bulk of the light-regulated genes with Group 1 showed relatively strong expression in B compared to R at both 6 h and 24 h, while Group 2 had very little to no response at 24 h to B. The two response patterns likely represent two distinct regulatory circuits, the first of which (Group 1) may be more dependent on input from cryptochromes, while the second (Group 2) is more dependent on phytochromes. It should be noted that phytochromes also absorb B light and therefore can mediate responses at these wavelengths (Franklin et al., 2003). It is therefore possible that the B light induction of Group 2 genes after 6 h could be due to activation of phytochrome A. However, as FR light was ineffective, this is unlikely to be a major contribution to the response.

Analysis of the genes in these sets (Figures 6, 7) gives some clues about the nature of these circuits with the different gene sets having different proportions of the two expression profiles. Group 1 chlorophyll biosynthesis genes contain most of the key regulatory targets in the tetrapyrrole pathway (Matsumoto et al., 2004; Stephenson and Terry, 2008) including *StHEMA1*, discussed earlier, but also other genes that show general light regulation in Arabidopsis (Matsumoto et al., 2004). The rest of the chlorophyll biosynthesis genes fall into Group 2. For PSII genes, it was noticeable that Group 2 genes included most of those associated with the oxygen-evolving complex and thus less critically dependent on light induction compared to antenna or core photosystem proteins (Supplementary Tables S12, S13). Similarly, other photosynthesis-related genes were more likely to be in Group 2, while in general the photosystem categories contained predominantly Group 1 genes. Overall, these data suggest that Group 1 contains genes that are more dependent on light regulation and Group 2, genes that are less dependent. It is therefore interesting that GA biosynthesis genes fall into the Group 1 pattern of expression suggesting that induction of GAs is not a secondary consequence of light exposure, but an important part of the immediate response to activation of light signaling pathways.

We also observed a third expression pattern when looking at tetrapyrrole (chlorophyll) biosynthesis genes. Group 3 genes showed no light induction after 24 h exposure to WL, B, and R. The tetrapyrrole biosynthesis pathway is important for synthesizing heme required throughout the cell, including for respiration, and a subset of tetrapyrrole genes has regularly been observed to show no light induction in Arabidopsis (Ujwal et al., 2002). These have been associated with the synthesis of extraplastidic hemes and include *HEMA2* and genes encoding ferrochelatase 1 (*FC1*), protoporphryrinogen IX oxidase (PPO) (Nagai et al., 2007; Moulin et al., 2008). These genes were also identified in group 3 in the current study. In Arabidopsis, this cohort of genes shows induction in response to stress, presumably because heme is required as a ligand for many proteins involved in responding to stress, and it would be interesting to test if this is the case in potato.

To identify TFs that might be involved in the regulation of gene expression we identified TFs that showed a similar expression pattern to Group 1 genes. As shown in Table 1, amongst the genes that progressively accumulated after light exposure were *S. tuberosum* homologues of *HY5*, the PSEUDO-RESPONSE REGULATORS, *PRR2, PRR5* and *PRR9*, and AP2/EREBP transcription factors. In Arabidopsis seedlings, *HY5* is a positive regulator of genes encoding chlorophyll *a*/*b* binding proteins (LHCB) and many photosynthesis proteins as well as flavonoid biosynthesis-related genes (Osterlund et al., 2000) whereas PRRs have been postulated as positive regulators of light signaling under the control of phyB (Takase et al., 2013). In addition, AP2/EREBP transcription factors are implicated in plant acclimation to environmental signals (Dietz et al., 2010). These results indicate that the light induction of genes encoding photosynthesis proteins as well as those involved in flavonoid, carotenoid, and GA biosynthesis in potato tubers is likely to be regulated by these same transcription factors found to be mediating light signaling responses in Arabidopsis seedlings.

In addition, it has been proposed that TFs functioning early in signaling pathways might show transient induction under those conditions (Khanna et al., 2006). We identified seven TF genes that were expressed more strongly at 6 h compared to 24 h (Table 2). This set of TFs included genes encoding Dof zinc finger family proteins that have been implicated as regulators of light responses (Le Hir and Bellini, 2013) providing further support for the hypothesis that light induction of chloroplast gene expression in potato tuber tissue is similar to pathways regulated by phytochrome and cryptochrome signaling in Arabidopsis seedlings. Taken together our transcriptome results for the light regulation of gene expression during tuber greening are consistent with data obtained in young Arabidopsis seedlings.

### Implications for Reducing Potato Waste

Our results have a number of implications for the practical reduction of potato waste due to greening. Tuber shelf life could be prolonged by packaging that can absorb B and R light wavelengths of light and transmit more of the FR wavelengths. In addition, providing supplementary FR to the lighting used for retail and storage of potatoes could also prolong the shelf life and thereby reduce waste. Finally, the observations here that the light signaling profile for the induction of chlorophyll and GA biosynthesis genes (Group 1) is very similar gives promise to the idea that tuber-targeted intervention of these signaling pathways could simultaneously block both the real (GA) and perceived (chlorophyll) problems that result in potato waste due to greening.

## Data Availability Statement

The datasets generated for this study can be found in the ArrayExpress at https://www.ebi.ac.uk/arrayexpress/experiments/E-MTAB-7707/.

## Author Contributions

HO, AW, VG, MJT, and MAT conceived and designed this project. AW and VG provided the materials. HO, LD, JA, and PH collected and analyzed the data. HO, LD, MJT, and MAT interpreted the data. HO, MJT and MAT wrote the manuscript.

## Conflict of Interest

AW and VG were employed by company Branston Ltd. The remaining authors declare that the research was conducted in the absence of any commercial or financial relationships that could be construed as a potential conflict of interest.

## Footnotes

1 http://www.ebi.ac.uk/arrayexpress/

2 http://systemsbiology.cau.edu.cn/agriGOv2/

3 https://ics.hutton.ac.uk/solarray

## Supplementary Material

FIGURE S1 Chlorophyll and carotenoid accumulation in KE tubers exposed to white light **(A)** tubers were exposed to white light intensities of 0.5, 5, 14, or 130 μmol/m^2^/s or kept in darkness for 7 days at 18°C and total chlorophyll and carotenoid accumulation was measured. Data shown are mean ± S.D. (*n* = 3 independent biological replicates). **(B)** Photographs of representative tubers after transfer into white light (14 μmol/m^2^/s) for the days indicated.

FIGURE S2 Light induction of chlorophyll and carotenoid accumulation in KE tubers **(A)** re-plotting of data in Figure 2A. Tubers were exposed to far-red (FR), blue (B), red (R), white light (WL) or kept in darkness (Dark) at 18°C for 1, 4, or 7 days and total chlorophyll was plotted as μg per gram dry weight (g.D.W.). **(B)** Chlorophyll *a* and *b* was measured separately in the samples shown in **(A)**. Data shown are mean ± S.D. (*n* = 3 independent biological replicates). Asterisks indicate statistical differences between day 0 and respective treatments as determined by Student’s *t*-test (*p* < 0.05^∗^, *p* < 0.001^∗∗^, *p* < 0.0001^∗∗∗^).

FIGURE S3 Sequence alignment of HEMA proteins a multiple amino acid sequence alignment of proteins encoded by three potato *HEMA* genes, StHEMA1, 2, and 3 together with Arabidopsis HEMA1 At1g58290 is shown. The functional amino acid domains found in the Arabidopsis HEMA1 and StHEMA proteins are underlined with the critical amino acids for catalysis and chloroplast import highlighted in red and green, respectively. The amino acid residues found critical in binding the final product, glutamate-1-semialdehyde in Arabidopsis are highlighted in orange. Asterisks show the residues conserved in all four proteins while the colons and periods indicate the conservation of position between amino acids with strongly and weakly similar properties, respectively.

FIGURE S4 Evolutionary relationship of HEMA and phytochrome sequences estimates of evolutionary divergence between HEMA **(A)** and phytochrome **(B)** amino acid sequences of Arabidopsis and several Solanaceae and other crop species. The number of amino acid differences per site from **(A)** HEMA sequences of Arabidopsis, tomato (*Solanum lycopersicum*), cucumber (*Cucumis sativus*), rice (*Oryza sativa*), and potato and **(B)** phytochrome sequences from Arabidopsis, tomato and potato, are shown. All positions containing gaps and missing data were eliminated. There were a total of 443 and 1066 positions for HEMA and phytochrome, respectively in the final dataset. Evolutionary analyses were conducted in MEGA7 (Kumar et al., 2016).

FIGURE S5 Sequence alignment of PSY proteins a multiple amino acid sequence alignment of two potato *PSY* genes, StPSY1 and StPSY2, together with Arabidopsis PSY (At5g17230) is shown. The functional amino acid domain for chloroplast import is underlined. Asterisks show conserved residues in all four proteins while the colons and periods indicate the conservation of position between amino acids with strongly and weakly similar properties, respectively.

FIGURE S6 Gene expression in the steroid branch of the mevalonate biosynthesis pathway tubers were exposed to white light (WL), blue (B), red (R), or far-red (FR), or kept in darkness (Dark) at 18°C for 1, 4, or 7 days and RNA was extracted. Expression of *LAS1* **(A)** and *SSR1/DWF1* **(B)** encoding lanosterol synthase 1 and sterol side-chain reductase 1, respectively, was determined by quantitative RT-PCR and is shown relative to Dark 0 days and normalized to *β-TUBULIN*. Data shown are mean ± S.D. (*n* = 3 independent biological replicates). **(C)** The steroid branch of the mevalonate biosynthesis pathway with relevant genes indicated.

FIGURE S7 The effect of supplementary far-red light on chlorophyll and GA biosynthesis gene expression tubers were exposed to white light (WL) or white and far-red (FR) light for 4 days and expression of chlorophyll **(A)** and GA **(B)** biosynthesis pathway genes was determined by quantitative RT-PCR and is shown relative to Dark 0 days and normalized to *β-TUBULIN*. Data shown are mean ± S.D. (*n* = 3 independent biological replicates). See Figures 3, 5 for pathway details.

FIGURE S8 Expression of phytochrome genes in KE tubers were exposed to red **(A)** or blue **(B)** light at 18°C for 4 or 7 days and RNA was extracted. Expression of *PHYA* and *PHYB* genes encoding phytochrome A and phytochrome B, respectively, was determined by quantitative RT-PCR and is shown relative to Dark 0 days and normalized to *β-TUBULIN*. Data shown are mean ± S.D. (*n* = 3 independent biological replicates).

FIGURE S9 Hierarchical relationship of gene ontology terms following light treatment in KE tubers hierarchical tree showing the over-represented AgriGO v2 GO terms for the genes differentially expressed on exposure to white light for **(A)** 6 and **(B)** 24 h, The Phureja DM1-3 PGSC gene ID for the entire genome was used as background, and the expression data analyzed using the Fisher test method, Yekuteli FDR, at a *p*-value ≤ 0.05, with a minimum of 10 mapping entries. Boxes in the graph represent GO terms labeled by their GO ID. Significant terms (adjusted *p*-value ≤ 0.05) are marked with color, while non-significant terms are shown as white boxes.

TABLE S1 List of genes significantly regulated by 6 h White light.

TABLE S2 List of genes significantly regulated by 6 h Blue light.

TABLE S3 List of genes significantly regulated by 6 h Red light.

TABLE S4 List of genes significantly regulated by 24 h White light.

TABLE S5 List of genes significantly regulated by 24 h Blue light.

TABLE S6 List of genes significantly regulated by 24 h Red light.

TABLE S7 Primer list.

TABLE S8 List of selected tetrapyrrole genes used to construct Figure 6E in the order of hierarchy shown on the heat map.

TABLE S9 List of selected glycoalkaloid genes used to construct Figure 6F in the order of hierarchy shown on the heat map.

TABLE S10 List of selected flavonoid genes used to construct Figure 6F in the order of hierarchy shown on the heat map.

TABLE S11 List of selected carotenoid genes used to construct Figure 6F in the order of hierarchy shown on the heat map.

TABLE S12 List of BIN1_PSII genes used to construct Figure 7A in the order of hierarchy shown on the heat map.

TABLE S13 List of BIN1_PSI genes used to construct Figure 7A in the order of hierarchy shown on the heat map.

TABLE S14 List of BIN1_ cytb6f and F-ATPase genes used to construct Figure 7C in the order of hierarchy shown on the heat map.

TABLE S15 List of BIN1_other photosynthesis genes used to construct Figure 7B in the order of hierarchy shown on the heat map.

TABLE S16 List of BIN1_Calvin-Benson cycle genes used to construct Figure 7B in the order of hierarchy shown on the heat map.

TABLE S17 List of BIN27.3_transcription factor genes that are more than 2-fold up-regulated in at least one of the 6 light conditions when compared to the dark control. Eighteen transcription factor genes that are consistently and progressively up-regulated at 6 h and 24 h are indicated in red text and are also shown in Table 1. Seven transcription factor genes that are up-regulated at 6 h and were down at 24 h are indicated in blue text and are also shown in Table 2.

TABLE S18 List of BIN27.3_transcription factor genes that are more than 2-fold down regulated in at least one of the 6 light conditions compared to the dark control.

DATA SHEET S1 Full method description for UHPLC-MS analysis of GAs and chromatograms for detected GAs.
