# Supplementary material for: Light Regulation of Chlorophyll and Glycoalkaloid Biosynthesis During Tuber Greening of Potato S. tuberosum
Source: Front Plant Sci. 2020 Jun 30;11:753. doi: 10.3389/fpls.2020.00753 (PMC7372192; doi:10.3389/fpls.2020.00753)
Supplement: FIGURE S1 — Chlorophyll and carotenoid accumulation in KE tubers exposed to white light (A) tubers were exposed to white light intensities of 0.5, 5, 14, or 130 μmol/m2/s or kept in darkness for 7 days at 18°C and total chlorophyll and carotenoid accumulation was measured. Data shown are mean ± S.D. (n = 3 independent biological replicates). (B) Photographs of representative tubers after transfer into white light (14 μmol/m2/s) for the days indicated. [file Data_Sheet_2.pdf]

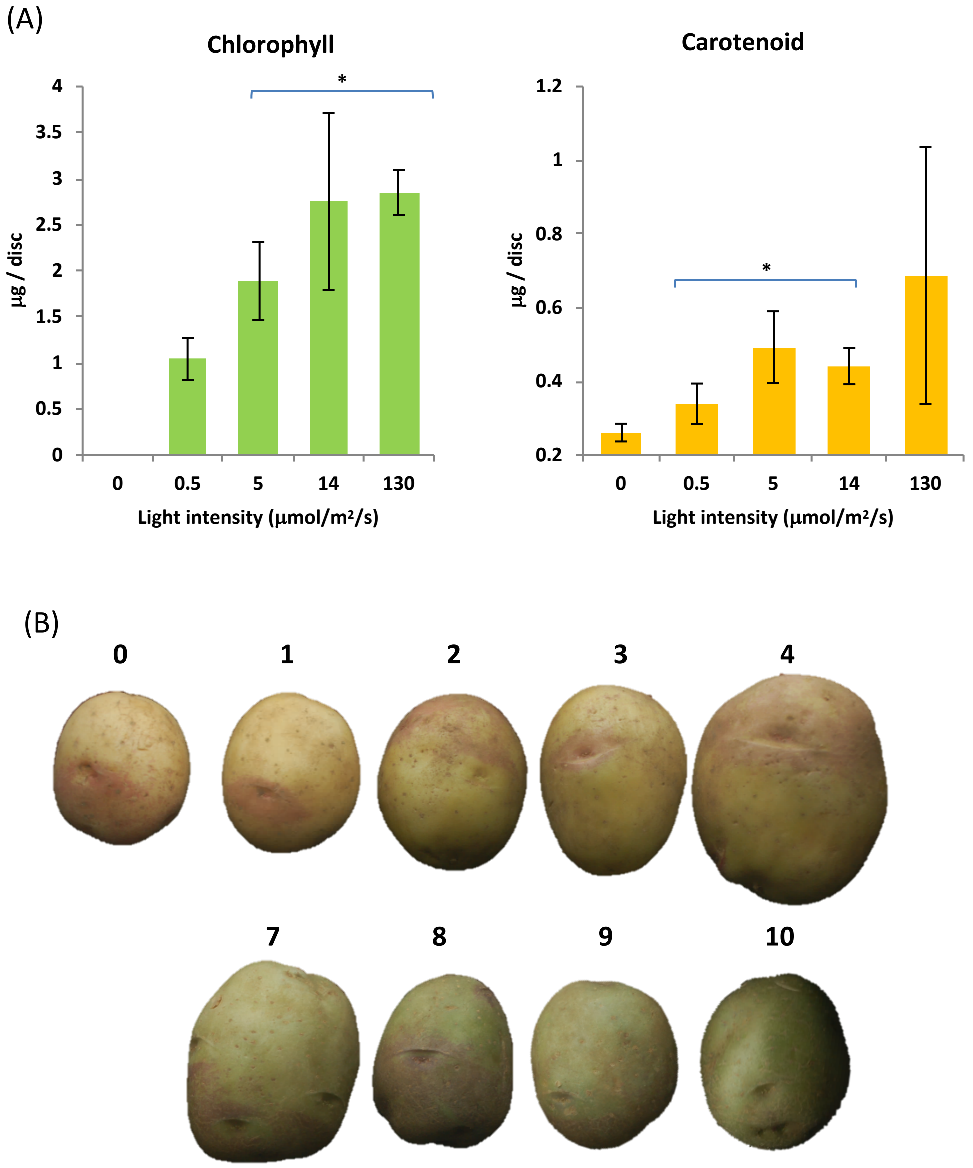

Figure S1 Chlorophyll and carotenoid accumulation in KE tubers exposed to white light (A) Tubers were exposed to white light intensities of 0.5, 5, 14, or 130 µmol/m^2/s or kept in darkness for 7 days at 18 °C and total chlorophyll and carotenoid accumulation was measured. Data shown are mean ± S.D. (n = 3 independent biological replicates). (B) Photographs of representative tubers after transfer into white light (14 µmol/m^2/s) for the days indicated. Asterisks indicate statistical differences between the samples indicated as determined by Student's *t*-test ($p < 0.05$*).

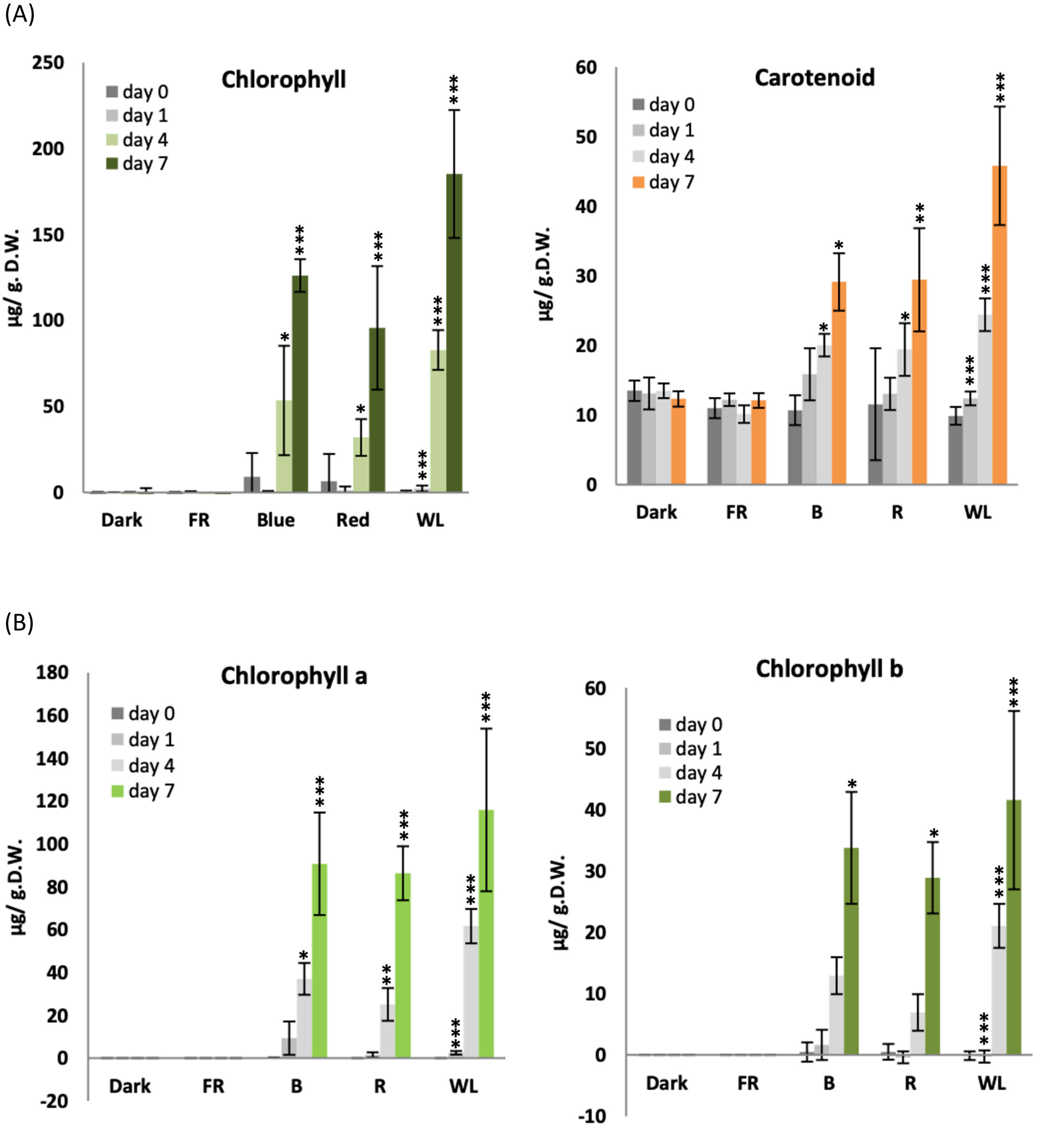

Figure S2 Light induction of chlorophyll and carotenoid accumulation in KE tubers (A) Re-plotting of data in Figure 2A. Tubers were exposed to far-red (FR), blue (B), red (R), white light (WL) or kept in darkness (Dark) at 18 °C for 1, 4, or 7 days and total chlorophyll was plotted as μg per gram dry weight (g.D.W.). (B) Chlorophyll *a* and *b* were measured separately in the samples shown in (A). Data shown are mean ± S.D. (n = 3 independent biological replicates). Asterisks indicate statistical differences between day 0 and respective treatments as determined by Student's *t*-test ($p < 0.05$*, $p < 0.001$**, $p < 0.0001$***).

S. tubelosum HEMA1 amino acid sequence alignment with Arabidopsis HEMA1

```
PGSC0003DMT400008033 StHEMA3     ------------------------------------------------------------
PGSC0003DMT400008034 StHEMA2     MAVASSFVTGGSFNC----DYLSSTSCSSSSSPSVDHQVRFFGSRRRIKPLVYWTRSHSL
A. thaliana HEMA1 At1g58290      MAVSSAFVGCPKLETLLNHHNLSPS--SSSSSSVSQTPLGLNGVRVLPKNNRTRRGLI-Q
PGSC0003DMT400016326 StHEMA1     MAIPTAFSGAK-LES---------L--SSSAAAASPASIGLFCNPGRVRRLSIQKGLFNS

PGSC0003DMT400008033 StHEMA3     -------------------------------------------MSSNIMVIGLNIHTAPV
PGSC0003DMT400008034 StHEMA2     SPKCELSSNSVVKNGK---KESRTSSLSALELLKTSAADRYTKESSSIMVIGLNIHTAPV
A. thaliana HEMA1 At1g58290      KARCELSA--------SSDSASNAASISALEQLKNSAADRYTKERSSIVVIGLSIHTAPV
PGSC0003DMT400016326 StHEMA1     GIRCEVAASDVVDQSQSSGNVTSSSSLSALEQLKSSAADRYTKERSSIVVIGLSIHTTPV
                                                                             *.*:****.***:**

PGSC0003DMT400008033 StHEMA3     ELREKVSIPESEWPQAIRELCSLNHIEEAAVLSTCNRIEIYVVALSKNRGIKEVTEWMCK
PGSC0003DMT400008034 StHEMA2     EVREKVSIPEAQWPQAIRELCSLNHIEEAAVLSTCNRIEIYVLALAQNRGIKEVTEWMSK
A. thaliana HEMA1 At1g58290      EMREKLAIPEAEWPRAIAELCGLNHIEEAAVLSTCNRMEIYVLALSQHRGVKEVTEWMSK
PGSC0003DMT400016326 StHEMA1     EVREKLAIPEAEWPRAIGELCNLNHIEEAAVLSTCNRMEIYVVALSQHRGVKEVTEWMSK
                                 *:***::***::**:** ***.***************:****:**:::**:*******.*

PGSC0003DMT400008033 StHEMA3     YSGVSVTELREHRFLLYNKDATQHLFEVAAGLDSLVLGEGQILAQVKQVVKNGQGERVPG
PGSC0003DMT400008034 StHEMA2     FSGVPVTELCQHRFLLYNQDATQHLFEVAAGLDSLVLGEGQILAQVKQVVKNGQG--VPG
A. thaliana HEMA1 At1g58290      TSGIPVSEICQHRFLLYNKDATQHIFEVSAGLDSLVLGEGQILAQVKQVVKVGQG--VNG
PGSC0003DMT400016326 StHEMA1     TSGVPVTEICKHRFLLYNNDATQHIFEVSAGLDSLVLGEGQILAQVKQVVKVGQG--VTG
                                 **: *:*: :*******:*****:***:********************** ***  * *

PGSC0003DMT400008033 StHEMA3     FGRKISELFKHAITAGKRVRTDTNISSGSVSVSSAAVELALLKHIPDSSSTSPRVLIVGA
PGSC0003DMT400008034 StHEMA2     FGRKISELFKRAITTGKRVRTETNISSGSVSVSSAAVELALLK-LPEYSSSTARVLIVGA
A. thaliana HEMA1 At1g58290      FGRNISGLFKHAITVGKRVRTETNIASGAVSVSSAAVELALMK-LPQSSNVSARMCVIGA
PGSC0003DMT400016326 StHEMA1     FGRNISGLFKHAITVGKRVRTETNIAAGAVSVSSAAVELALMK-LPESCHTTARMLVIGA
                                 ***:** ***:***.******:***::*:************:* :*: .  : *: ::**

PGSC0003DMT400008033 StHEMA3     GKMGKLVIGHLIAKGCKEMVVVNRTADRVAAIREELNTDNAGVIIEYKPFSEMLSCAAQA
PGSC0003DMT400008034 StHEMA2     GKMGKLVIRHLVAKGCKKMVVVNRTADRVAAIREELMDA----DIVYKPFSELLACAAQS
A. thaliana HEMA1 At1g58290      GKMGKLVIKHLMAKGCTKVVVVNRSEERVSAIREEMP----GIEIIYRPLDEMLACASEA
PGSC0003DMT400016326 StHEMA1     GKMGKLVIKHLVAKGCTKMVVVNRSEDRVSAIREEMK----DVEIIYKPLNEMLKCAAEA
                                 ******** **:****.::*****: :**:*****:        * *:*:.*:* **:::

PGSC0003DMT400008033 StHEMA3     DVIFTCTASKDPLFVKESVQAALRCV-NSSRSRLFIDISVPRNVESSVSELEGACIYNVD
PGSC0003DMT400008034 StHEMA2     DVIFTCTASKAPLFIKESVQALPSVNSEDGCRRLFIDISVPRNVEPSVAELEGAGVYNVD
A. thaliana HEMA1 At1g58290      DVVFTSTASETPLFLKEHVENLPQASPEVGGLRHFVDISVPRNVGSCVGEVETARVYNVD
PGSC0003DMT400016326 StHEMA1     DVIFTSTASETPLFMKERVVDLPPVGASVGSLRLFIDISVPRNVGACVNELEDARVYNVD
                                 **:**.***: ***:** *         . .  * *:********  .* *:* * :****

PGSC0003DMT400008033 StHEMA3     NLNEVVEANKEDRLRKKKEAEVIIAEEVKKFEALKDSLETVPTIKKLRACAERIRAAEVE
PGSC0003DMT400008034 StHEMA2     DLKEVVEANKEDRLRKKMEAETIIAEEVKQFEALKDSLETVPTIKKLRAYAERIRSAEVD
A. thaliana HEMA1 At1g58290      DLKEVVAANKEDRMRKAMEAQTIITEESTQFEAWRDSLETVPTIKKLRAYAERIRVAELE
PGSC0003DMT400016326 StHEMA1     DLKEVVAANKEDRLRKAMEAQAIISEESKQFEAWRDSLETVPTIKKLRAYAERIRVAELE
                                 :*:*** ******:**  **:.**:** .:*** :************** ***** **::

PGSC0003DMT400008033 StHEMA3     KCLSKIGDDDLSENNKNAIYDLSLAIINKMLHGPMKHLKCDGTQNRNLSDILENMHALNR
PGSC0003DMT400008034 StHEMA2     KCLSKMGDD-IPQNKKKAIYDLSLGIVNKLLHGPMQHLKCDGAENRTLSEILENMHALNR
A. thaliana HEMA1 At1g58290      KCMSKMGDD-INKKTTRAVDDLSRGIVNRFLHGPMQHLRCDGSDSRTLSETLENMHALNR
PGSC0003DMT400016326 StHEMA1      KCMSKMGDD-ISKKTKKAVDDLSRGIVNKLLHGPMQHLRCDGSDSRTLSETLENMHALNR
                                 **:**:*** : ::...*: *** .*:*::*****:**:***::.*.**: *********

PGSC0003DMT400008033 StHEMA3     IFDLDTEMD---DKLQAKLQENQKQSS*
PGSC0003DMT400008034 StHEMA2     IFGLDTEMSVLEEKVRAKIEQNQKQSS*
A. thaliana HEMA1 At1g58290      MYGLEKDIL--EEKLKAMAEQQQK----
PGSC0003DMT400016326 StHEMA1     MFSLETDISVLEQKIRAKVEQTQK*---
                                 ::.*:.::    :*::*  :: **
```

Figure S3 Sequence alignment of HEMA proteins A multiple amino acid sequence alignment of proteins encoded by three potato *HEMA* genes, StHEMA1, 2, and 3 together with Arabidopsis HEMA1 At1g58290 is shown. The functional amino acid domains found in the Arabidopsis HEMA1 and StHEMA proteins are underlined with the critical amino acids for catalysis and chloroplast import highlighted in red and green, respectively. The amino acid residues found critical in binding the final product, glutamate-1-semialdehyde in Arabidopsis are highlighted in orange. Asterisks show the residues conserved in all four proteins while the colons and periods indicate the conservation of position between amino acids with strongly and weakly similar properties, respectively.

(A)

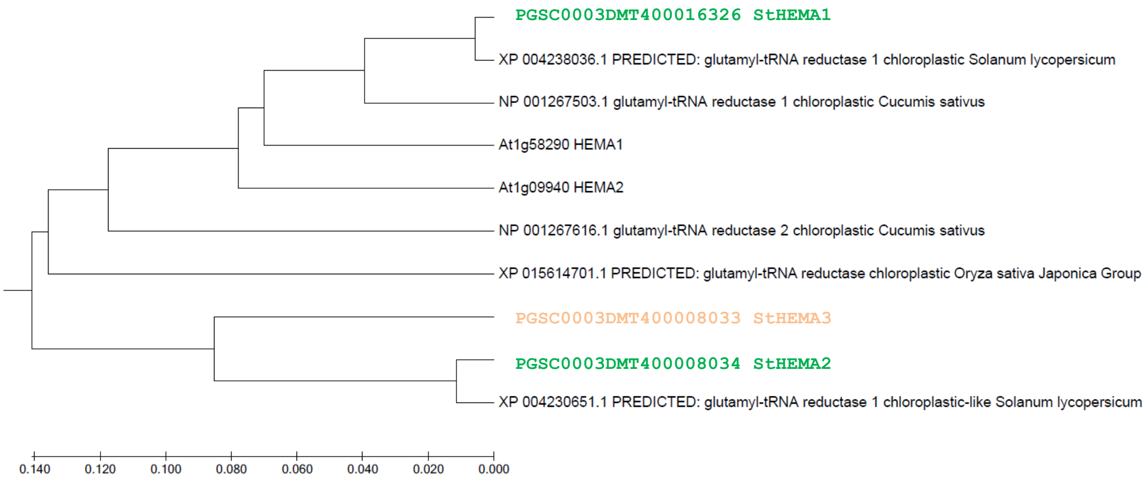

(B)

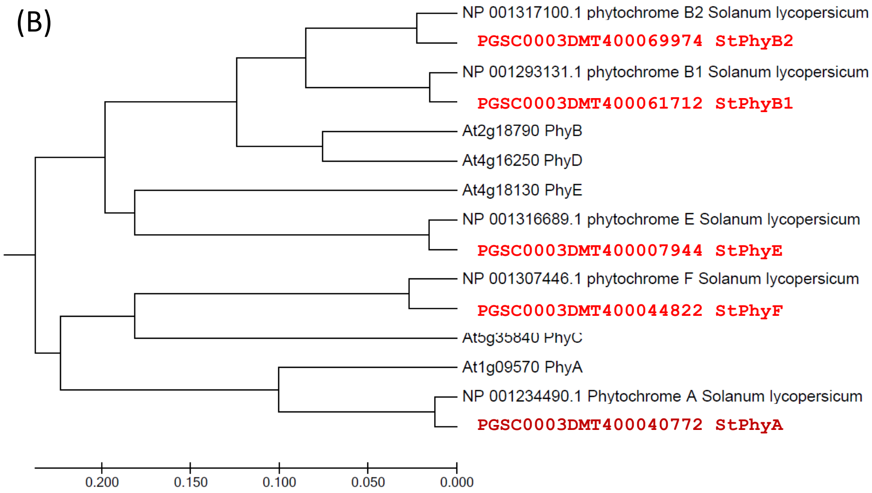

Figure S4 Evolutionary relationship of HEMA and phytochrome sequences
Estimates of evolutionary divergence between HEMA (A) and phytochrome (B) amino acid sequences of Arabidopsis and several Solanaceae and other crop species.
The number of amino acid differences per site from (A) HEMA sequences of Arabidopsis, tomato (*Solanum lycopersicum*), cucumber (*Cucumis sativus*), rice (*Oryza sativa*), and potato and (B) phytochrome sequences from Arabidopsis, tomato and potato, are shown. All positions containing gaps and missing data were eliminated. There were a total of 443 and 1066 positions for HEMA and phytochrome, respectively in the final dataset. Evolutionary analyses were conducted in MEGA7 (Kumar et al., 2016).

S. tubelosum PSY amino acid sequence alignment with Arabidopsis PSY

```
A. thaliana PSY At5g17230      MSSSVAVLWVATSSLNPDPMNNCGLVRV-------LESSRLFSPCQN----QRLNKGKKK 49
PGSC0003DMT400061846 StPSY1    --MSVALLWVVSPC---EVSNGTGFLESVREGKSFFDSSRHRNL----VSNERI------ 45
PGSC0003DMT400043103 StPSY2    --MSVALLWVVSPN--SEVLNGTGFLDSVREGNRGLESSRFPSPNRNSMWKGRFKKG--- 53
                                  ***:***.:     :  *. *::         ::***  .         *:

A. thaliana PSY At5g17230      QIPTWSSSFVRN------------RSRRIGVVSSSLVASPSGEIALSSEEKVYNVVLKQA 97
PGSC0003DMT400061846 StPSY1    ----------------NRGGGKQTNNGRKFSVRSAIVATPSGERTMTSEQMVYDVVLRQA 89
PGSC0003DMT400043103 StPSY2    GRQEWNFGFLNADLRYSCLGRSRTENGRSFSVQSSLVASPAGEMAVSSEKKVYEVVLKQA 113
                                                       .. *   * *::**:*:** :::**: **:***:**

A. thaliana PSY At5g17230      ALVNKQLRSSSYDLDVKKPQDVVLPGSLSLLGEAYDRCGEVCAEYAKTFYLGTLLMTPER 157
PGSC0003DMT400061846 StPSY1    ALVKRQLRSTD---ELEVKPDIPVPGNLGLLSEAYDRCGEVCAEYAKTFNLGTMLMTPER 146
PGSC0003DMT400043103 StPSY2    ALVKRHLISTE---DIEVKPDIVVPGNLGLLSEAYDRCGEVCAEYAKTFYLGTMLMTPDR 170
                               ***:::* *:.   :::   *: :**.*.**.***************** ***:****:*

A. thaliana PSY At5g17230      RKAIWAIYVWCRRTDELVDGPNASHITPMALDRWEARLEDLFRGRPFDMLDAALADTVAR 217
PGSC0003DMT400061846 StPSY1    RRAIWAIYVWCRRTDELVDGPNASYITPAALDRWEDRLEDVFNGRPFDMLDGALSDTVSN 206
PGSC0003DMT400043103 StPSY2    RRAIWAIYVWCRRTDELVDGPNASHITPQALDRWEARLEDIFNGRPFDMLDAALSDTVSK 230
                               *:**********************:*** ****** ****:*.********.**:***:.

A. thaliana PSY At5g17230      YPVDIQPFRDMIEGMRMDLKKSRYQNFDDLYLYCYYVAGTVGLMSVPVMGIDPKSKATTE 277
PGSC0003DMT400061846 StPSY1    FPVDIQPFRDMIEGMRMDLRKSRYKNFDELYLYCYYVAGTVGLMSVPIMGIAPESKATTE 266
PGSC0003DMT400043103 StPSY2    FPVDIQPFRDMVEGMRMDLWKSRYNNFDELYLYCYYVAGTVGLMSVPIMGIAPESKATTE 290
                               :**********:******* ****:***:******************:*** *:******

A. thaliana PSY At5g17230      SVYNAALALGIANQLTNILRDVGEDARRGRVYLPQDELAQAGLSDEDIFAGKVTDKWRNF 337
PGSC0003DMT400061846 StPSY1    SVYNAALALGIANQLTNILRDVGEDARRGRVYLPQDELAQAGLSDEDIFAGRVTDKWRIF 326
PGSC0003DMT400043103 StPSY2    SVYNAALALGIANQLTNILRDVGEDARRGRVYLPQDELAQAGLSDEDIFAGRVTDKWRIF 350
                               ***************************************************:****** *

A. thaliana PSY At5g17230      MKMQLKRARMFFDEAEKGVTELSAASRWPVWASLLLYRRILDEIEANDYNNFTKRAYVGK 397
PGSC0003DMT400061846 StPSY1    MKKQIHRARKFFDDAEKGVTELSAASRFPVWASLVLYRKILDEIEANDYNNFTRRAYVSK 386
PGSC0003DMT400043103 StPSY2    MKKQIQRARKFFDEAEKGVTELSSASRWPVLASLLLYRKILDEIEANDYNNFTRRAYVSK 410
                               ** *::*** ***:*********:***:** ***:***:**************:****.*

A. thaliana PSY At5g17230      VKKIAALPLAYAKSVLKTSSSRLSI----  422
PGSC0003DMT400061846 StPSY1    SKKLIALPIAYAKSLVPPTRTISLLS*--  412
PGSC0003DMT400043103 StPSY2    PKKLLTLPIAYARSLVPPKSTSSPLAKT*  438
                                **: :**:***:*::  . :   :
```

Figure S5 Sequence alignment of PSY proteins

A multiple amino acid sequence alignment of two potato *PSY* genes, StPSY1 and StPSY2, together with Arabidopsis PSY (At5g17230) is shown. The functional amino acid domain for chloroplast import is underlined. Asterisks show conserved residues in all four proteins while the colons and periods indicate the conservation of position between amino acids with strongly and weakly similar properties, respectively.

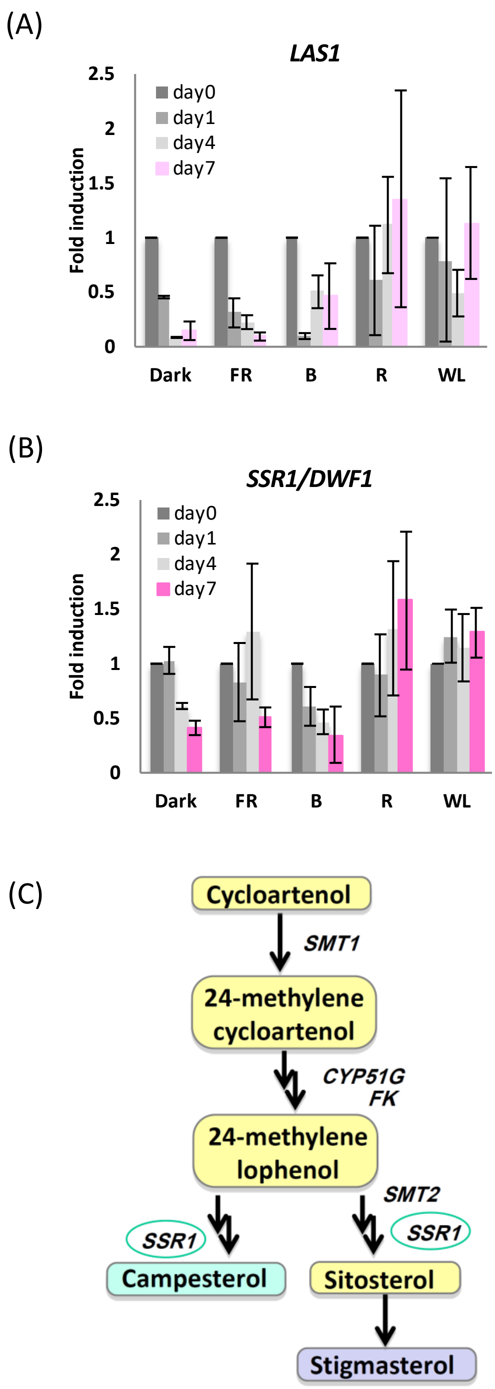

Figure S6 Gene expression in the steroid branch of the mevalonate biosynthesis pathway Tubers were exposed to white light (WL), blue (B), red (R), or far-red (FR), or kept in darkness (Dark) at 18 °C for 1, 4, or 7 days and RNA was extracted. Expression of *LAS1* (A) and *SSR1/DWF1* (B) encoding lanosterol synthase 1 and sterol side-chain reductase 1, respectively, was determined by quantitative RT-PCR and is shown relative to Dark 0 days and normalized to *β-TUBULIN*. Data shown are mean ± S.D. (n = 3 independent biological replicates). (C) The steroid branch of the mevalonate biosynthesis pathway with relevant genes indicated.

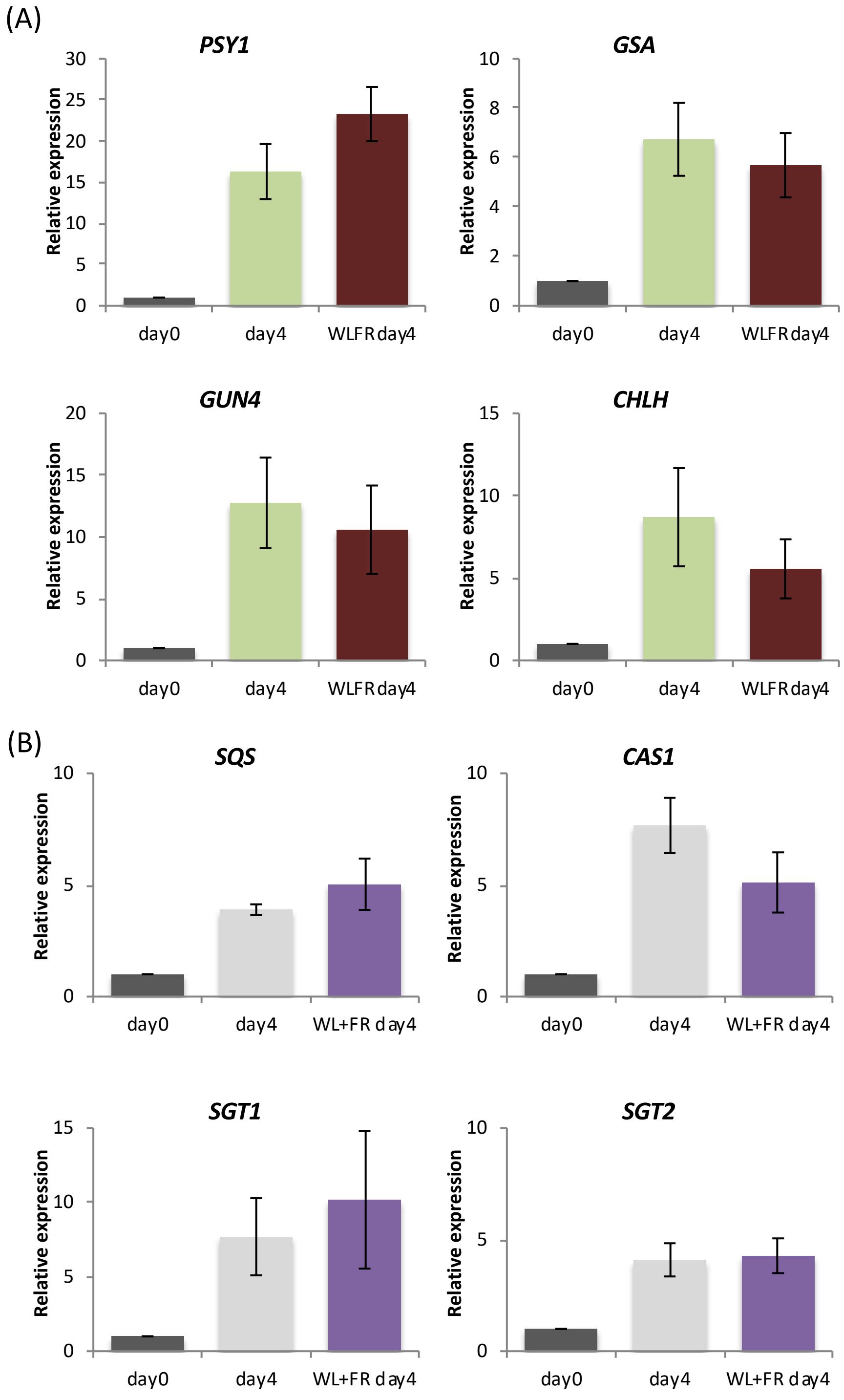

Figure S7 The effect of supplementary far-red light on chlorophyll and GA biosynthesis gene expression Tubers were exposed to white light only (day 4) or white and far-red (WL + FR day 4) light for 4 days and expression of chlorophyll (A) and GA (B) biosynthesis pathway genes was determined by quantitative RT-PCR and is shown relative to Dark 0 days and normalized to *β-TUBULIN*. Data shown are mean ± S.D. (n = 3 independent biological replicates). See Figures 3 and 5 for pathway details.

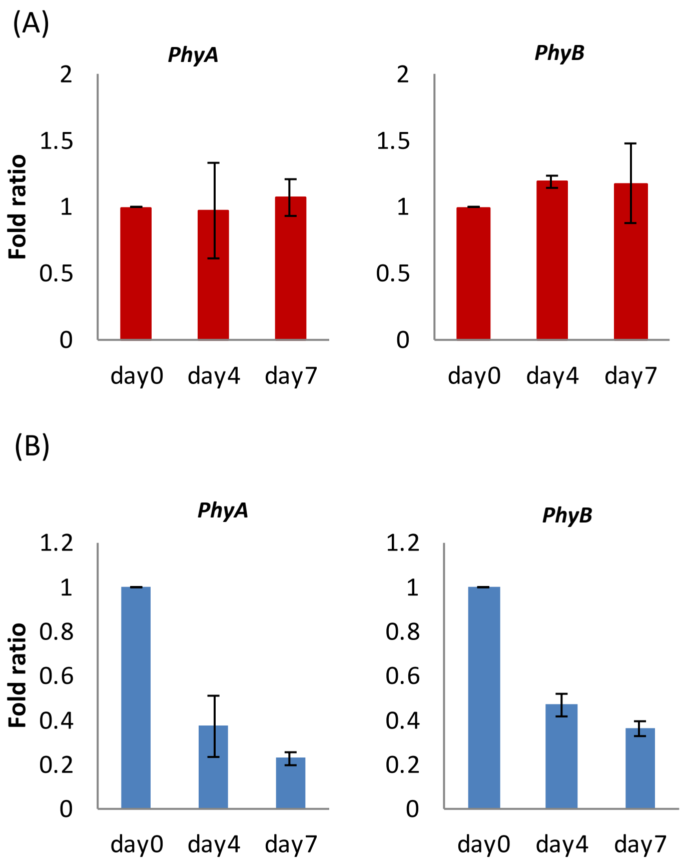

Figure S8 Expression of phytochrome genes in KE tubers Tubers were exposed to red (A) or blue (B) at 18 °C for 4, or 7 days and RNA was extracted. Expression of *PHYA* and *PHYB* genes encoding phytochrome A and phytochrome B, respectively, was determined by quantitative RT-PCR and is shown relative to Dark 0 days and normalized to *β-TUBULIN*. Data shown are mean ± S.D. (n = 3 independent biological replicates).

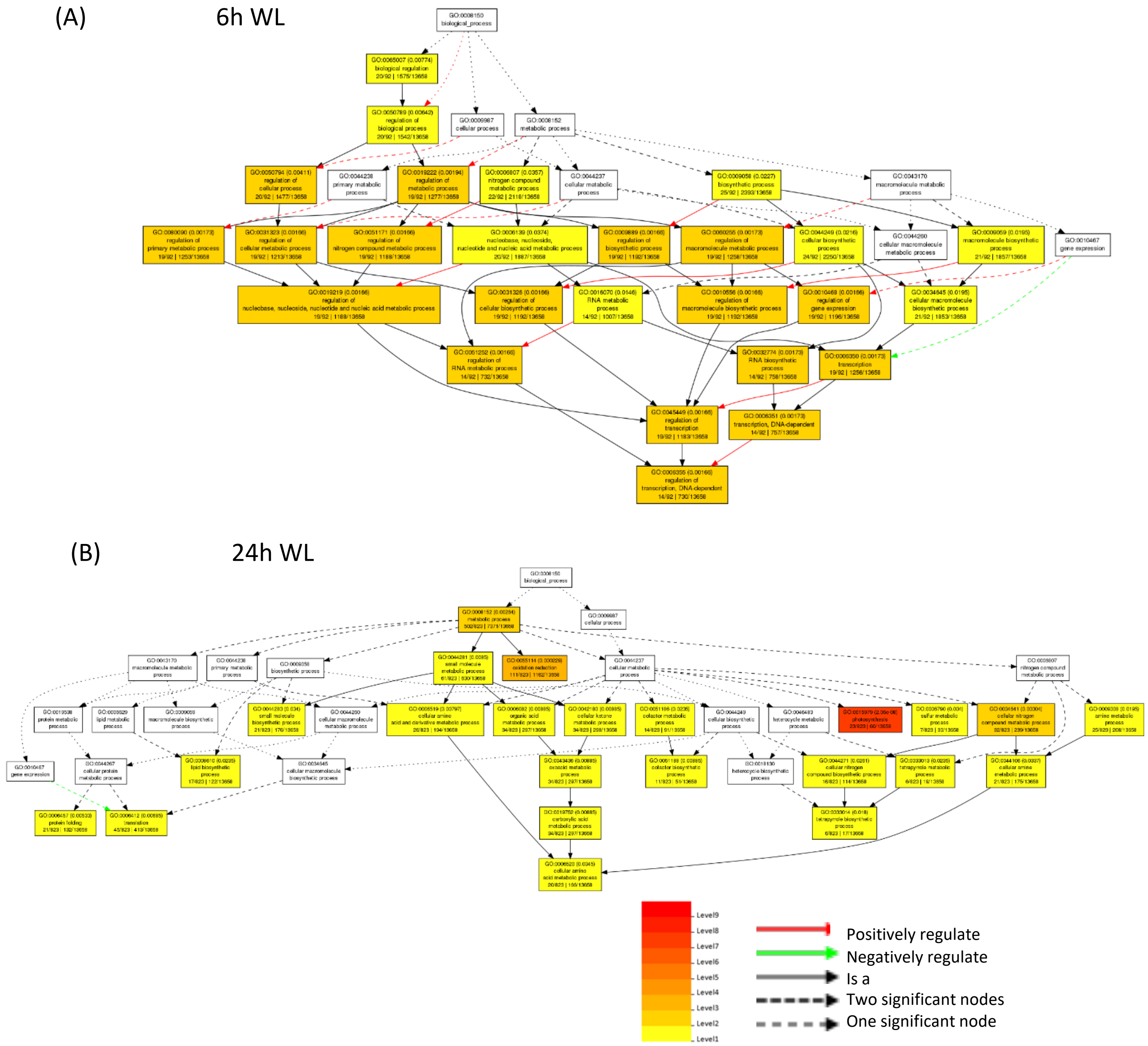

Figure S9 Hierarchical relationship of changes in gene ontology terms following light treatment in KE tubers Hierarchical tree showing the over-represented AgriGO v2 GO terms for the genes differentially expressed on exposure to white light for 6 (A) and 24 (B) hours, The Phureja DM1-3 PGSC gene ID for the entire genome was used as background, and the expression data analysed using the Fisher test method, Yekuteli FDR, at a *p*-value ≤0.05, with a minimum of 10 mapping entries. Boxes in the graph represent GO terms labelled by their GO ID. Significant terms (adjusted *p*-value ≤0.05) are marked with colour, while non-significant terms are shown as white boxes.
