# Supplementary material for: Light Regulation of Chlorophyll and Glycoalkaloid Biosynthesis During Tuber Greening of Potato S. tuberosum
Source: Front Plant Sci. 2020 Jun 30;11:753. doi: 10.3389/fpls.2020.00753 (PMC7372192; doi:10.3389/fpls.2020.00753)

Full method description for UHPLC-MS analysis of GA's

UHPLC separations were performed with an Agilent 1260 Infinity HPLC system (Agilent, Cheadle U.K) operated at a flow rate of 400 µL/min, the column (Thermo Hypersil Gold C18, 50x2.1 mm, 1.9µm particle size (PN 25002-052130) and guard cartridge (3 µm particle size, 10x2.1 mm) fitted with a Uniguard cartridge holder (PN 25003-012101 and PN 852-00, Thermo Fisher Scientific, U.K.) were maintained at a temperature of 30 ^{o}C. The solvent A, HPLC grade water, and solvent B, HPLC grade acetonitrile (JT Baker U.K.) were acidified with 0.2% [v/v] formic acid (P/N A117-50, Fisher Scientific U.K.). The gradient programme was as follows: 5-15% B 0-3 min, 15-25% B 3-7 min, 25-50% B 7-10 min, hold 50% B 10-13 minutes, 50-100% B 13-14 min, hold 100% B 14-16 min, 100-5% B 16-17 min, equilibration 5% B 17-22 min. Autosampler syringe and line washes were performed with 8:2 HPLC grade acetonitrile:water (JT Baker, UK).

The HPLC eluent was next transfered to the Agilent 6230 TOF/MS system equipped with an Agilent Jet stream dual ESI source and operated under Agilent Mass Hunter software (Agilent, Cheadle, U.K.). The TOF/MS was operated in ESI positive 4 GHz high resolution mode within the 'normal' scan range 100-3000 *m/z*. The TOF/MS system was tuned and calibrated following the manufacturers recommended procedures, achieving a mass resolution of 25,000> (FWHM defined from *m/z* 500-3000) and mass accuracy within +/- 0.0001 *m/z*. The eluent was diverted to waste from 0-0.8 mins, to the MS detector from 0.8-16 mins, and back to waste from 16-22 mins. Mass spectra were primarilly collected between *m/z* 80-2000, a scan rate of 1.37 spectra/sec was applied. The following settings were applied to the TOF/MS: MCP 720 V; PMT 632 V; Amp Offset 36036 DAC; Pusher 1250 V; Puller -800 V; Puller offset 28 V; Acc focus: -1920 V; Front mirror -6500 V; Mid mirror -1386 V; Back mirror 1650 V. The following settings were applied to, Optics 1: Fragmentor 175 V; Skimmer 65 V; Oct 1 RF 750 V, and Optics 2: Ion focus -80 V; Slicer -8 V; Horizontal Q 26.5 V; Vert. Q 26.7 V; Top slit 17 V; Bottom slit 16.4 V; energy offset 0 V. The following settings were applied to ESI: Gas Temp 350°C; Drying gas flow 9L/min; Nebuliser 40 PSIG; Sheath gas temp 380°C; Sheath gas flow 11L/min; Cap V 4000; Nozzle V 1000. To maintain high levels of mass accuracy (+/- 3ppm), the TOF/MS was operated with the manufacturers recommended reference solution delivered via a secondary HPLC pump at a flow rate of 2.5 mL/min applying a 1:100 flow splitter (25 ul/min) to a secondary ESI nebuliser, 121.050873 *m/z* and 922.009798 *m/z* were applied as lock masses.

The samples were analysed in a completely randomised order. For each analytical block, initially six injections of a QC sample (equal mixture of each individual extract) were performed for system conditioning. After which, three further injections of QC samples were made and followed by 5-6 experimental samples and a further QC injection. This process was repeated until all experimental samples were analysed, finally two QC injections were made at the end of the analytical block. A control blank sample was analysed at the start and end of the analytical block. Reference samples

were also extracted and analysed in triplicate. The QC sample provides a measure of analytical error, the reference provides a measure of technical (extraction) and analytical error. High purity standards of alpha-chaconine (PN 80075) and alpha-solanine (PN 80074) were obtained from LGC limited (Teddington, Middlesex, U.K.), an equimolar (250 µM) solution was diluted in 1:1 HPLC grade methanol:water (250>125>62.5>31.25>15.625>7.8125>3.9063>1.9531>0.9766> 0.4883 µM), and analysed under identical conditions.

Potato LC-ESI+ MS Total Ion Current (TIC) Chromatogram (A) and Extracted Ion Chromatograms (EIC) of glyco-alkaloids (B).

(A)

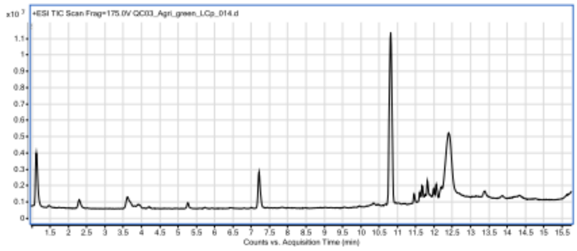

(B)

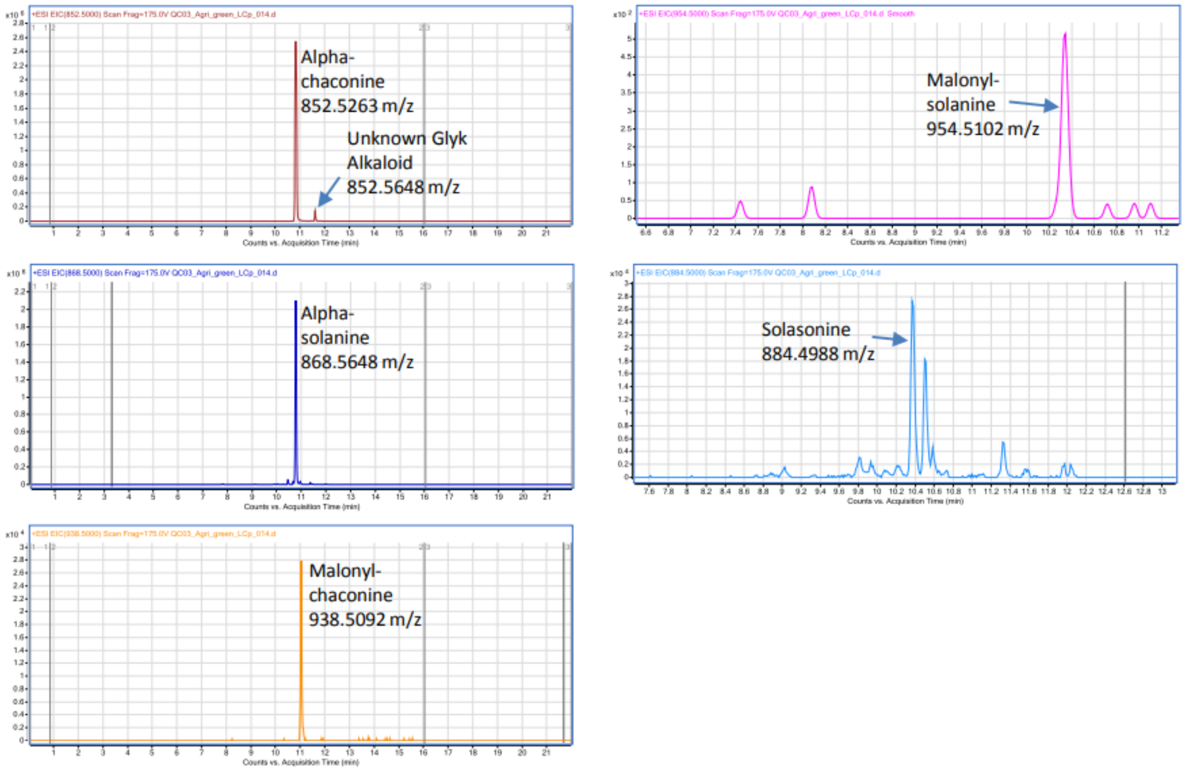

Supplement: DATA SHEET S1 — Full method description for UHPLC-MS analysis of GAs and chromatograms for detected GAs. [file Data_Sheet_1.PDF]
